# Supplementary figures and images for: A MademoiseLLE domain binding platform links the key RNA transporter to endosomes
Source: PLoS Genet. 2022 Jun 21;18(6):e1010269. doi: 10.1371/journal.pgen.1010269 (PMC9249222; doi:10.1371/journal.pgen.1010269)

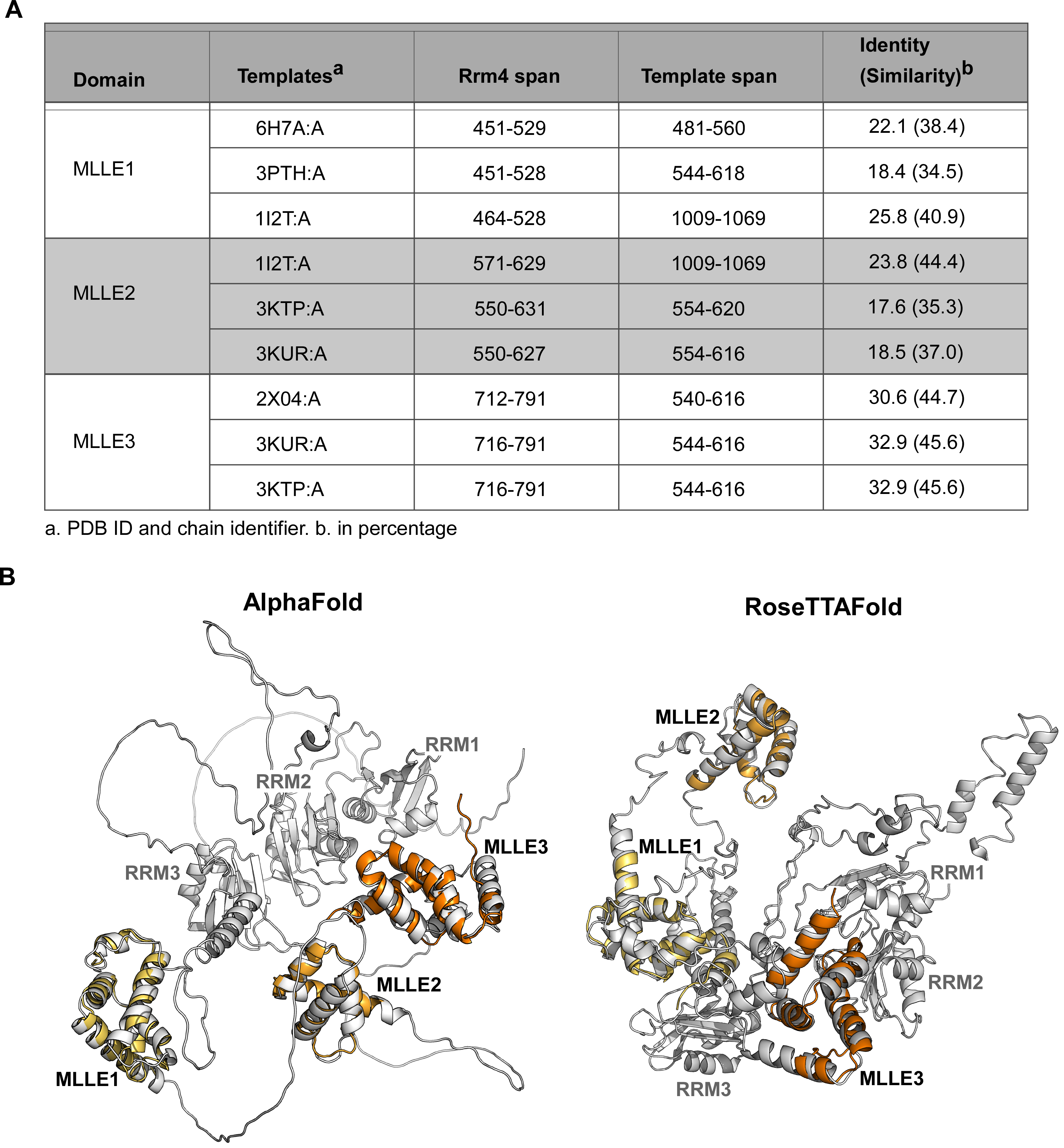

Supplement: S1 Fig — (A) Compilation of MLLE sequences used for modelling with the highest similarity of MLLE1-3Rrm4. (B) Structural models obtained with TopModel overlaid to Rrm4 full-length models obtained with the recently available tools as indicated. Natural alignments between corresponding MLLE domains have an RMSD < 2Å, mutually confirming the quality of the independently modelled structures. The differences in the relative domain arrangements in both full-length models and the disordered regions in between the domains suggest a high mobility within Rrm4. (TIF) [file pgen.1010269.s001.tif]

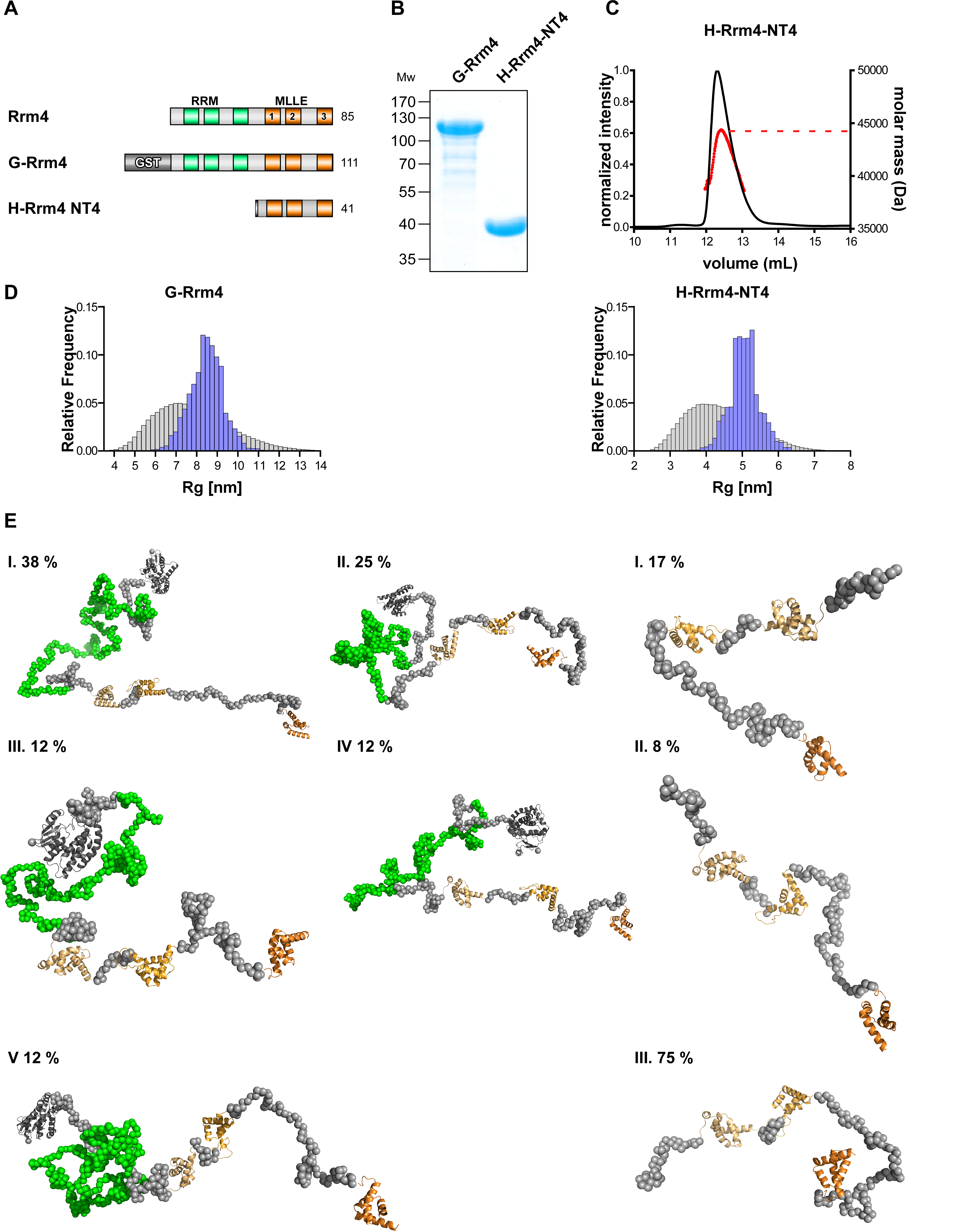

Supplement: S2 Fig — (A) Schematic representation of protein variants drawn to scale (molecular weight in kilo Dalton indicated next to protein bar) using the following coloring: dark green, RNA recognition motif (RRM); orange, MLLERrm4 domains; (B) SDS PAGE analysis of purified G-Rrm4, H-Rrm4-NT4 used in crystallography and SAXS measurement. (C) MALS-SEC analysis of H-Rrm4-NT4. Graph shows the elution profile. Dotted line in red indicate the apparent molecular weight as observed in the light scattering. (D) Rg distribution calculated by EOM pool is shown in grey bars and the selected models in blue bars left GST_Rrm4 right H-Rrm4NT4 (E) Left Selected models of the EOM analysis for GST-Rrm4. The MLLE subdomains and the GST are shown in cartoon representation (MLLE1 in light orange, MLLE2 in orange, MLLE 3 in dark orange) and the missing amino acids as grey spheres. I: The model has a Rg of 8.94 nm, a Dmax of 29.22 nm with a volume fraction of~0.38. II: The model has a Rg of 8.75 nm, a Dmax of 23.99 with a volume fraction of~0.25. III: The model has a Rg of 7.74 nm, a Dmax of 25.90 with a volume fraction of~0.12. IV: The model has a Rg of 8.33 nm, a Dmax of 28.79 with a volume fraction of~0.12. V: The model has a Rg of 9.14 nm, a Dmax of 33.73 with a volume fraction of~0.12. Right Selected models of the EOM analysis for H-Rrm4NT4. The MLLE subdomains are shown in cartoon representation (MLLE1 in light orange, MLLE2 in orange, MLLE3 in dark orange) and the missing amino acids as grey spheres. I: The model has a Rg of 5.12 nm, a Dmax of 15.56 with a volume fraction of~0.17. II: The model has a Rg of 5.90 nm, a Dmax of 18.73 nm with a volume fraction of~0.08. III: The model has a Rg of 5.10 nm, a Dmax of 16.43 nm with a volume fraction of~0.75. (TIF) [file pgen.1010269.s002.tif]

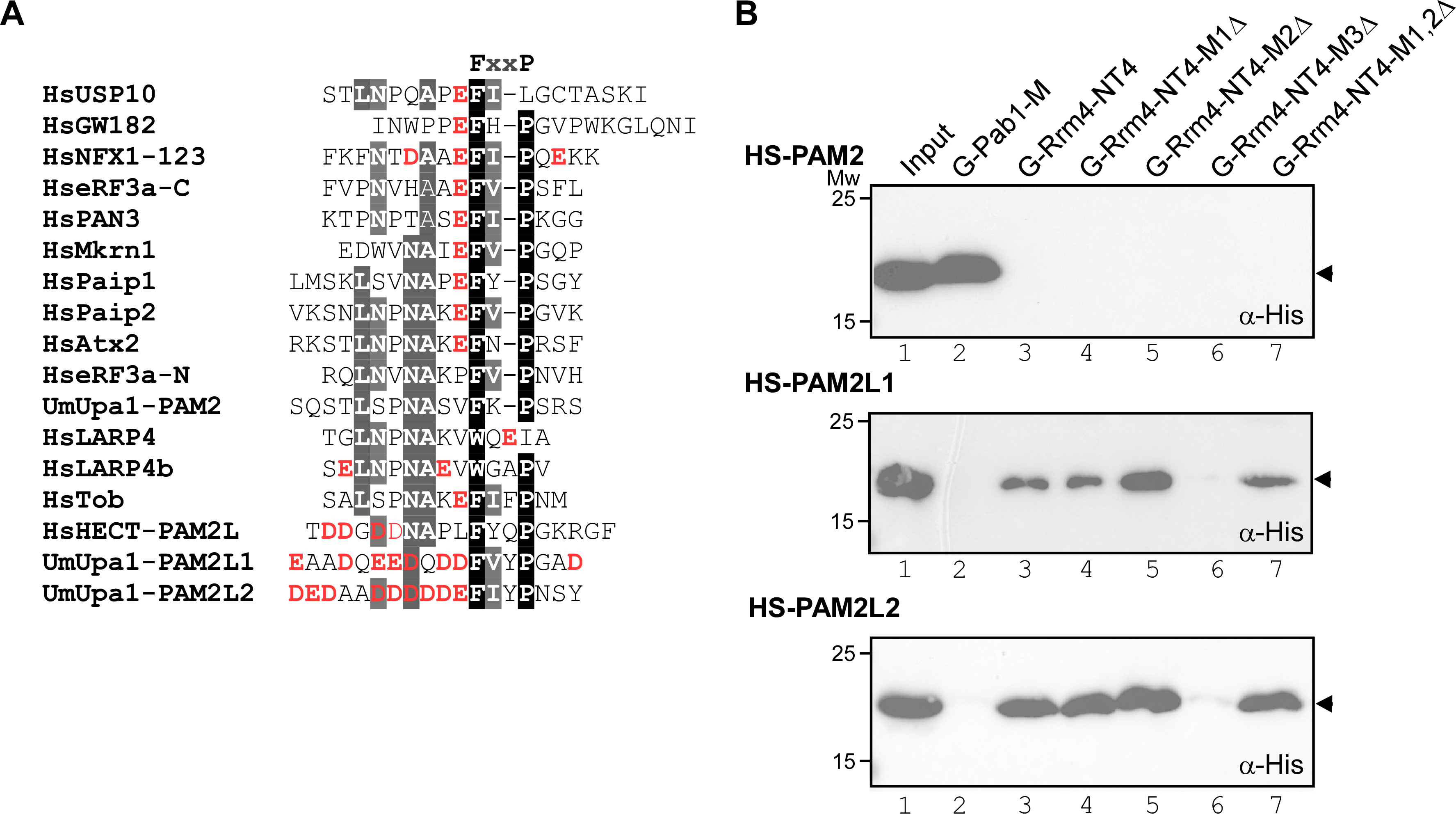

Supplement: S3 Fig — (A) Comparison of PAM2 sequences found in Upa1 (UniprotKB ID A0A0D1E015) with those of human proteins, such as Usp10 (Q14694), GW182 Q9HCJ0), Mkrn1 (Q9UHC7), Paip1 (Q9H074), Paip2 (Q9BPZ3), Atx2 (-Q99700), NFX (Q12986), eRF3 (P15170), PAN3 (Q58A45), LARP4 (Q71RC2), LARP4b (Q92615), Tob (P50616), HECT (O95071), Asp and Glu are indicated in red stressing the highly negative charges in PAM2L sequences. (B) Western blot analysis of GST co-purification experiments with components expressed in E. coli: N-terminal Hexa-Histidine-SUMO-tagged PAM2 variants were pulled down by N-terminal GST fused MLLE variants of Rrm4 and Pab1. Experiment was performed with the soluble fraction of E. coli cell lysate to demonstrate specific binding. Results were analysed with αHis immunoblotting. (TIF) [file pgen.1010269.s003.tif]

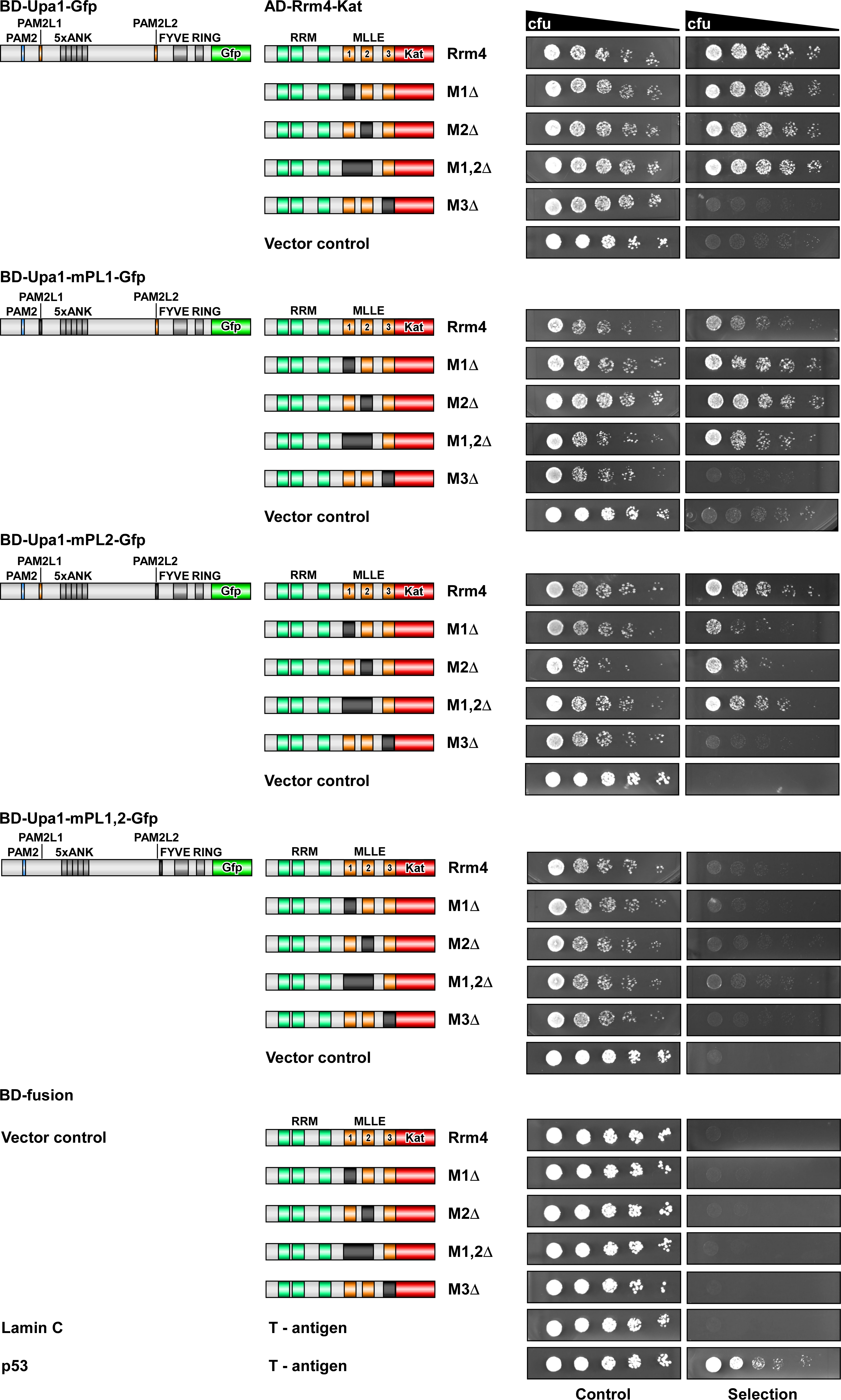

Supplement: S4 Fig — Yeast two-hybrid analyses with schematic representation of protein variants tested on the left. Cultures were serially diluted 1:5 (decreasing colony-forming units, cfu) and spotted on respective selection plates controlling transformation and assaying reporter gene expression (see Materials and methods). (TIF) [file pgen.1010269.s004.tif]

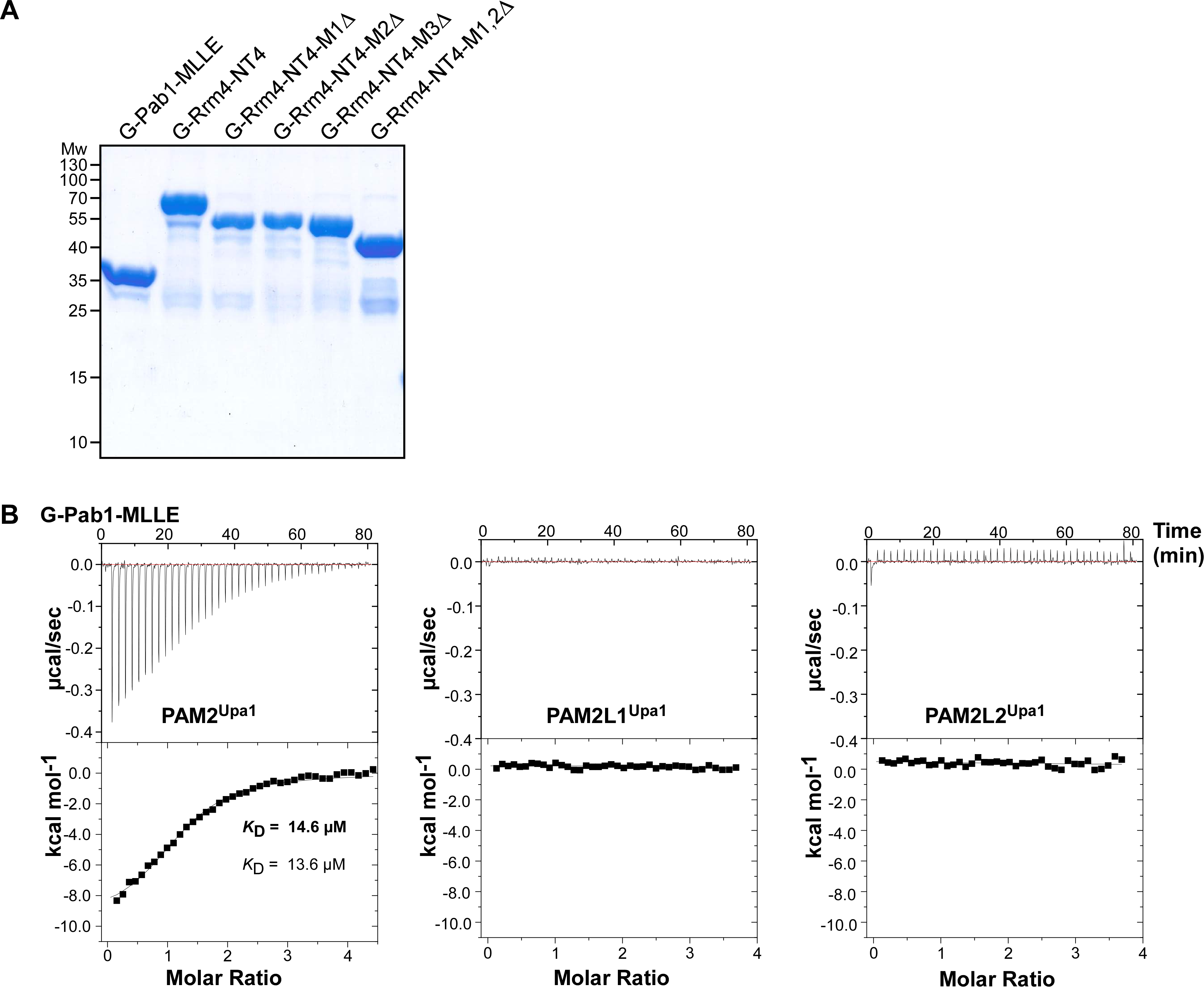

Supplement: S5 Fig — (A) SDS-PAGE analysis of purified GST-MLLE variants used in ITC experiments (see also S6 Fig). (B) Representative isothermal titration calorimetry (ITC) binding curves of MLLEPab1 domain. Experiments were performed using GST or hexa-histidine-tagged MLLE variants and synthetic PAM2 and PAM2L peptide variants. KD values of two independent measurements are given (values corresponding to the indicated data are given in bold). (TIF) [file pgen.1010269.s005.tif]

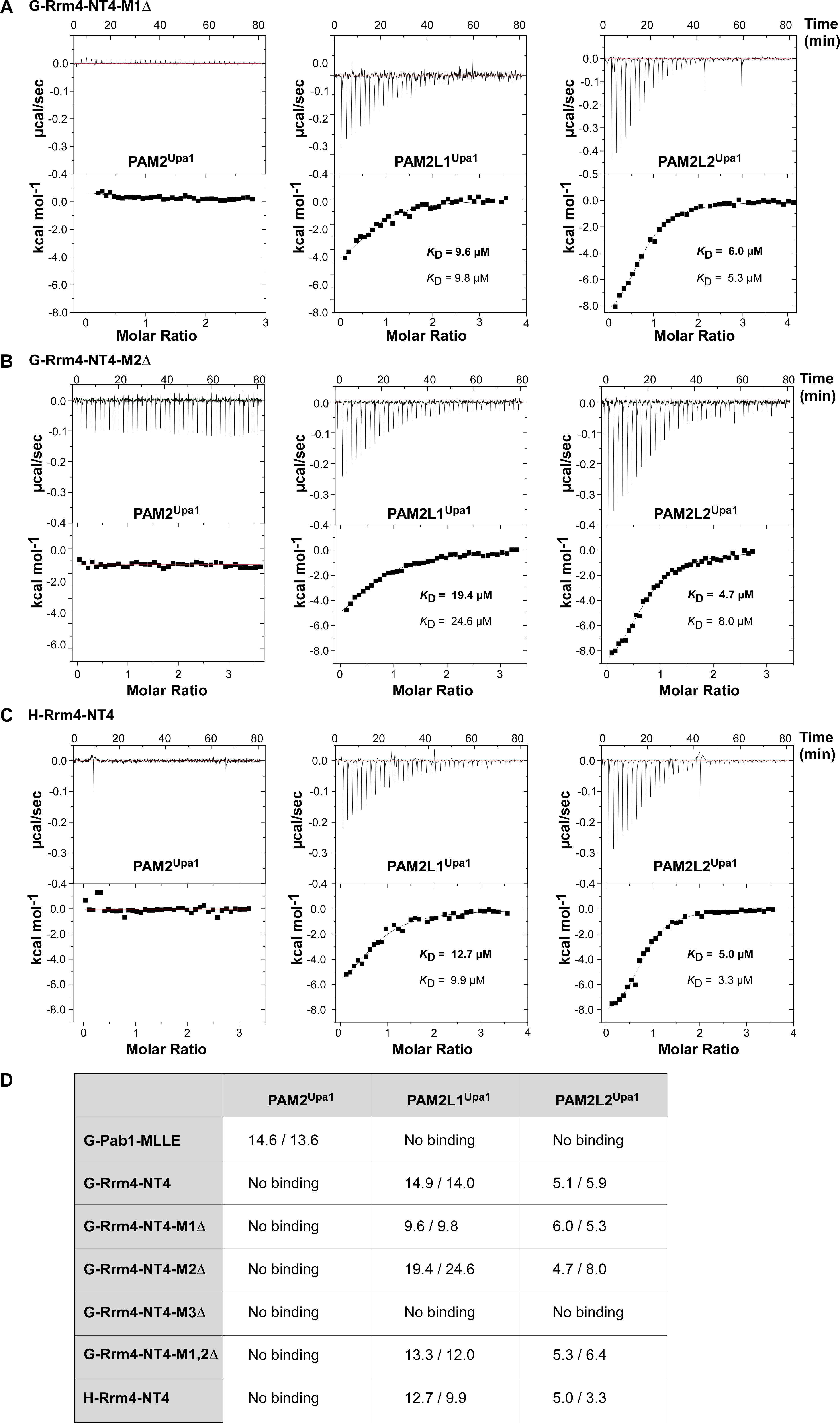

Supplement: S6 Fig — (A-C) Representative isothermal titration calorimetry (ITC) binding curves of MLLE domains. Experiments were performed using GST or hexa-histidine-tagged MLLE variants and synthetic PAM2 and PAM2L peptide variants. KD values of two independent measurements are given (values corresponding to the indicated data are given in bold). (D) Summary of ITC results shown in Figs 3 and S3. KD values are given in μM. (TIF) [file pgen.1010269.s006.tif]

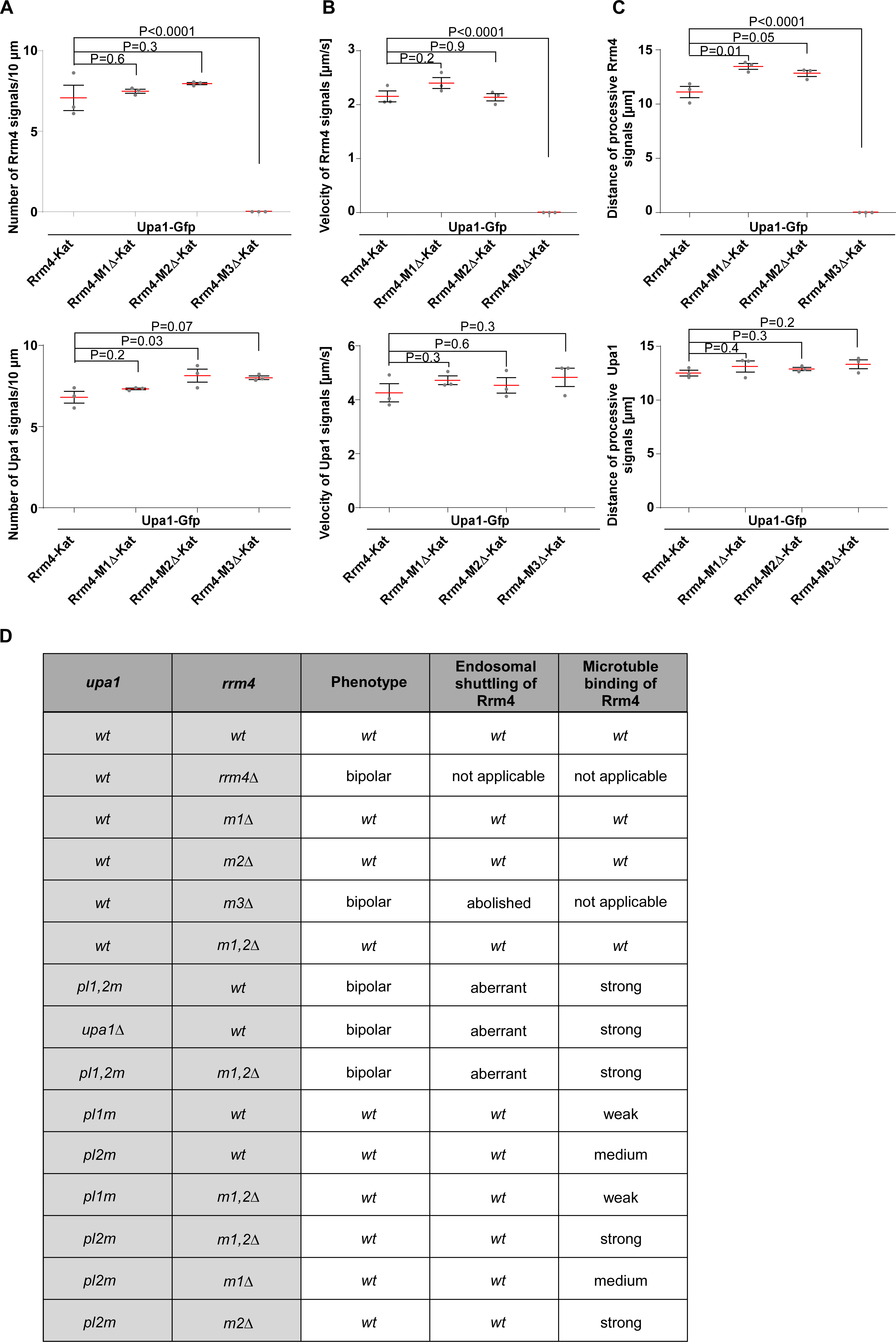

Supplement: S7 Fig — (A-C) Quantification of processive Rrm4-Kat (top) and Upa1-Gfp signals (bottom; (A)), velocity of fluorescent Rrm4-Kat (top) and Upa1-Gfp signals (bottom; (B)) and the travelled distance of processive Rrm4-Kat (top) and Upa1-Gfp signals (bottom; (C); per 10 μm of hyphal length; only particles with a processive movement of > 5 μm were conducted; data points representing mean from n = 3 independent experiments, with mean of means, red line and SEM; unpaired two-tailed Student’s t-test (α<0.05), for each experiment at least 25 hyphae were analysed per strain). (D) Summary of the in vivo analysis is shown in Figs 4,5 and S7–S10. (TIF) [file pgen.1010269.s007.tif]

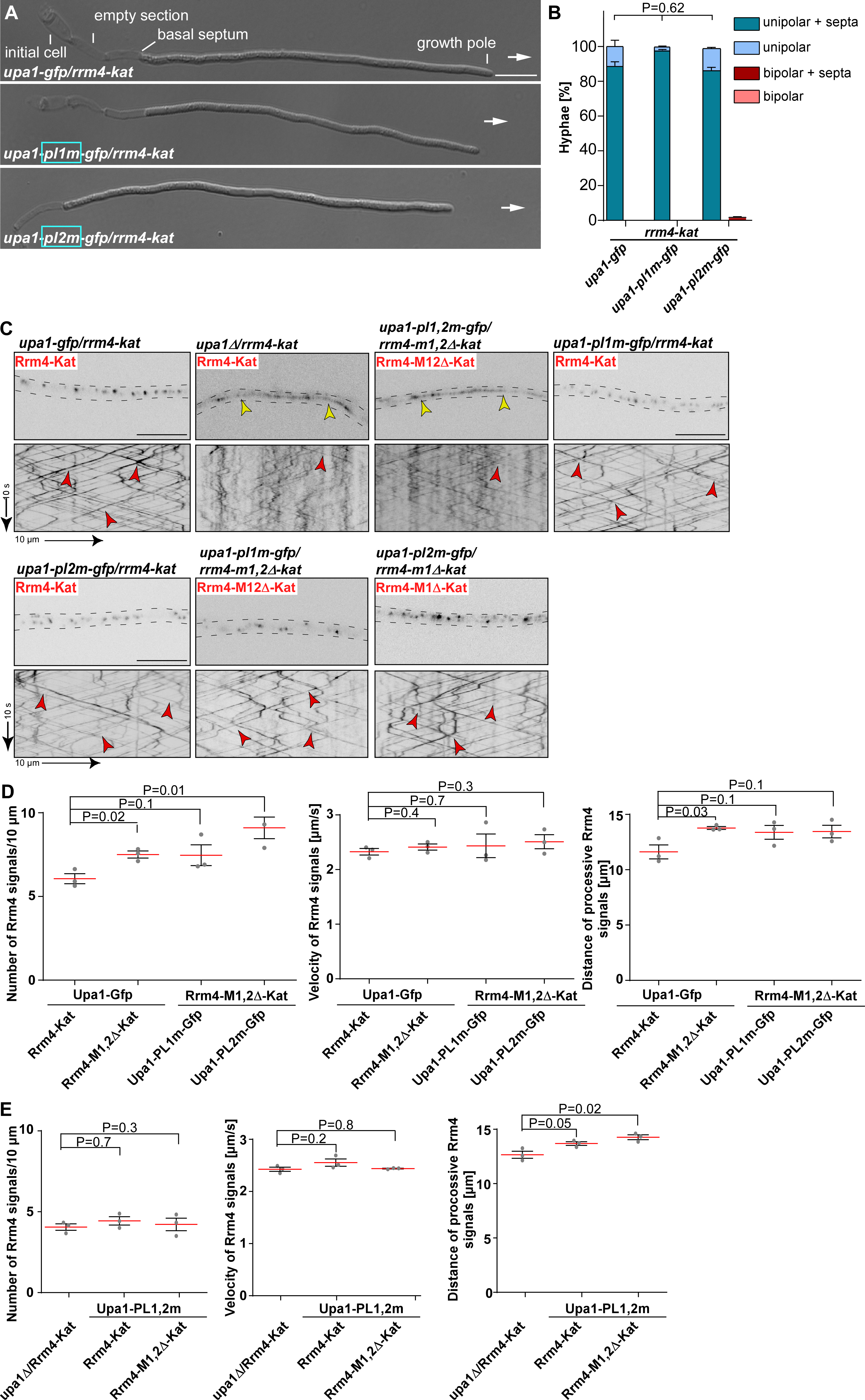

Supplement: S8 Fig — (A) Growth of AB33 derivatives in their hyphal form (6 h.p.i.; size bar 10 μm). Growth direction is marked by arrows. (B) Quantification of hyphal growth of AB33 derivatives shown in panel A (6 h.p.i.): unipolarity, bipolarity and basal septum formation were quantified (error bars, SEM.; n = 3 independent experiments, > 100 hyphae were analysed per strain; For statistical evaluation, the percentage of uni- and bipolarity was investigated by using unpaired two-tailed Student’s t-test (α<0.05). (C) Micrograph and Kymograph of AB33 hyphae derivates (6 h.p.i.) expressing red and green fluorescent proteins as indicated. Fluorescence signals were detected simultaneously using dual-view technology (arrow length on the left and bottom indicates time and distance, respectively). Processive co-localising signals are marked by red arrowheads. Aberrant microtubule staining is indicated by a yellow arrowhead. (D-E) Quantification of processive Rrm4-Kat signals (left), velocity of fluorescent Rrm4-Kat (middle) and the travelled distance of processive Rrm4-Kat signals (right) related to Fig 5C and EV5C, respectively (per 10 μm of hyphal length; only particles with a processive movement of > 5 μm were conducted; data points representing mean from n = 3 independent experiments, with mean of means, red line and SEM; unpaired two-tailed Student’s t-test (α<0.05), for each experiment at least 25 hyphae were analysed per strain). (TIF) [file pgen.1010269.s008.tif]

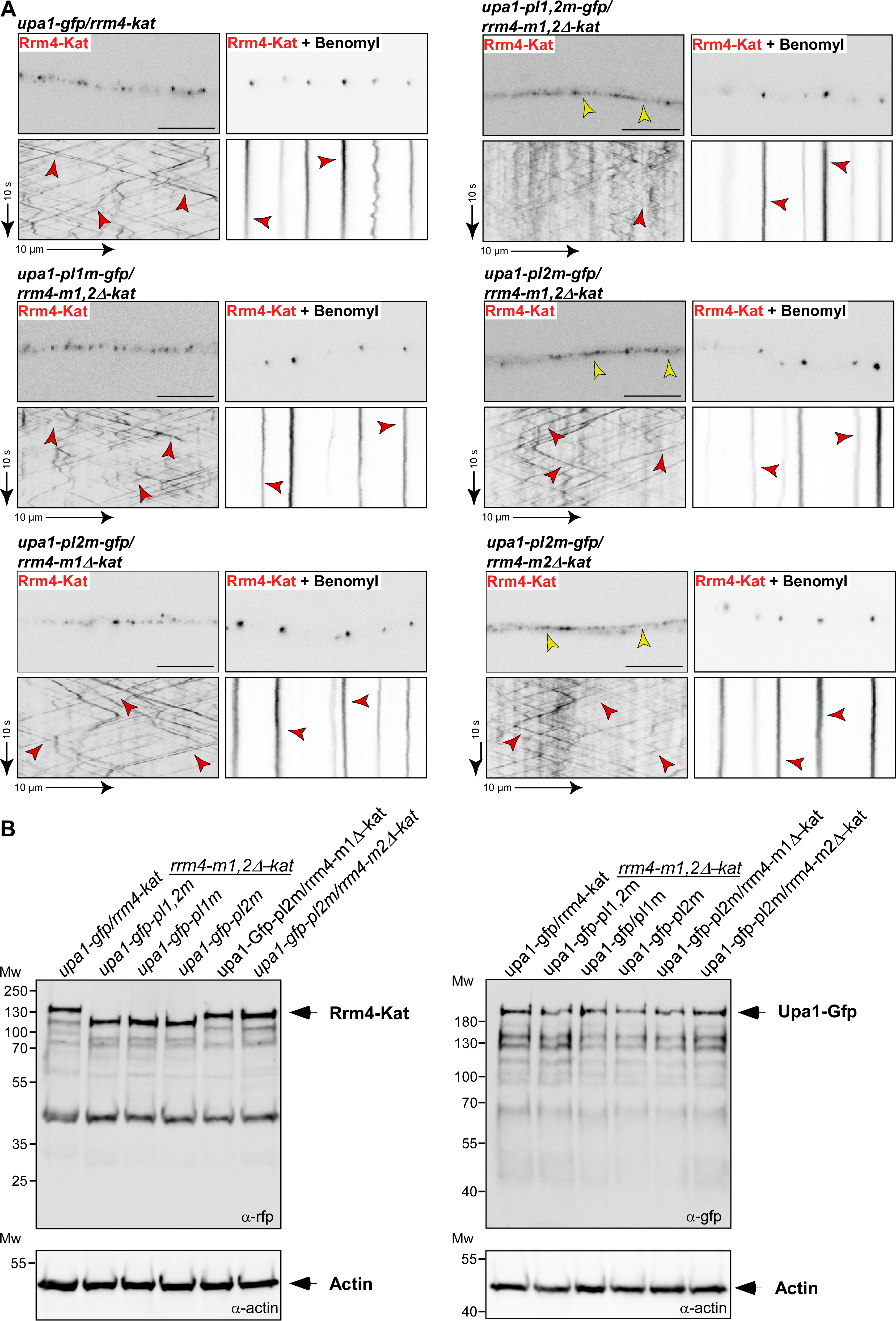

Supplement: S9 Fig — (A) Benomyl treatment is shown in micrograph and kymograph of AB33 hyphae derivates (6 h.p.i.) expressing red and green fluorescent proteins. Processive signals, as well as static signals, post benomyl treatment are marked by red arrowheads. Aberrant microtubule staining is indicated by a yellow arrowhead. (B) Western blot analysis of the expression levels of Rrm4 and Upa1 variants 6 h.p.i. of hyphal growth. Rrm4 and Upa1 variants were detected via mKate2 and Gfp, respectively. Actin was detected as loading control. Bands representing full-length proteins are marked with arrows. (TIF) [file pgen.1010269.s009.tif]

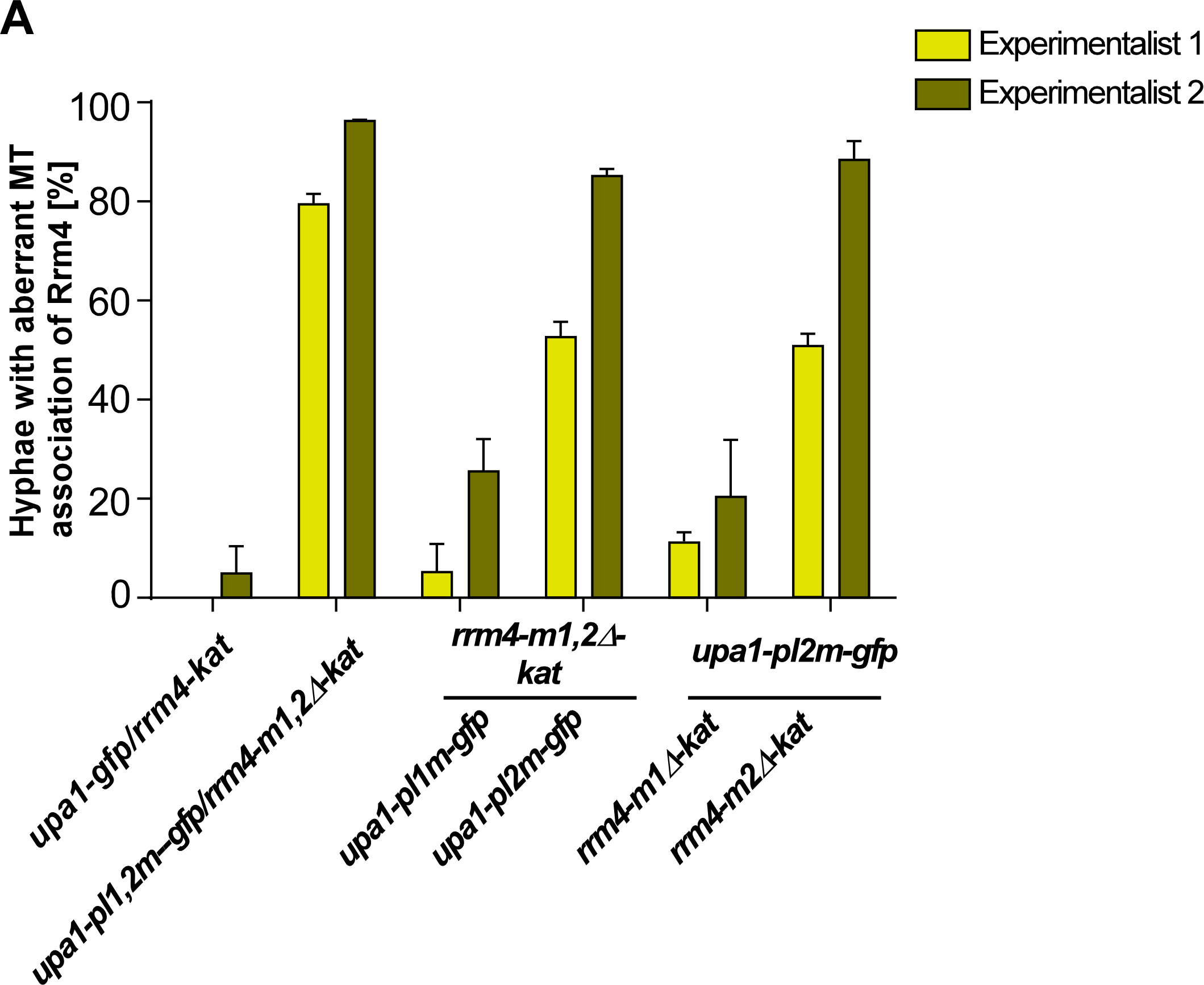

Supplement: S10 Fig — Evaluation of the most important strains showing aberrant microtubule staining analysed by two experimentalists (we used the data obtained by the more experienced microscopist in Fig 5D; see Materials and methods). (TIF) [file pgen.1010269.s010.tif]
