# Supplementary material for: A MademoiseLLE domain binding platform links the key RNA transporter to endosomes
Source: PLoS Genet. 2022 Jun 21;18(6):e1010269. doi: 10.1371/journal.pgen.1010269 (PMC9249222; doi:10.1371/journal.pgen.1010269)
Supplement: S1 Table — (RTF) [file pgen.1010269.s011.rtf]

S1 Table: Accession numbers for protein sequences used in multiple sequence alignment of MLLE domains
Organism Name	Protein Name	Domain Name	Uniprot KB Number	Sequence coverage	
Homo sapiens	Poly[A] binding protein, PABP	MLLEPABP	P11940	554 - 617	
Triticum aestivum	Poly[A] binding protein, PABP	MLLEPABP	P93616	564 - 627	
Trypanosoma cruzi	Poly[A] binding protein, PABP	MLLEPABP	Q27335	484 - 547	
Leishmania major	Poly[A] binding protein, PABP	MLLEPABP	E9AFX7	494 - 557	
Saccharomyces cerevisiae	Poly[A] binding protein, PABP	MLLEPABP	P04147	501 - 566	
Homo sapiens	E3 ubiquitin-protein ligase UBR5, EDD	MLLEUbr5	O95071	2390 - 2452	
Rattus norvegicus	E3 ubiquitin-protein ligase UBR5	MLLEUbr5	Q62671	2380-2442	
Ustilago maydis	Poly[A] binding protein, Pab1	MLLEPab1	Q4P8R9	567 - 630	
Ustilago maydis	Rrm4	MLLE3Rrm4	A0A0D1DWZ5	727 - 792	
Ustilago maydis	Rrm4	MLLE2Rrm4	A0A0D1DWZ5	564 - 629	
Ustilago maydis	Rrm4	MLLE1Rrm4	A0A0D1DWZ5	462 – 528	
