# Supplementary material for: A MademoiseLLE domain binding platform links the key RNA transporter to endosomes
Source: PLoS Genet. 2022 Jun 21;18(6):e1010269. doi: 10.1371/journal.pgen.1010269 (PMC9249222; doi:10.1371/journal.pgen.1010269)
Supplement: S2 Table — (RTF) [file pgen.1010269.s012.rtf]

S2 Table: Data collection and refinement statistics
	MLLE2Rrm4	
Wavelength	0.979340	
Resolution range	33.51  - 2.6 (2.693  - 2.6)	
Space group	P 43 21 2	
Unit cell	53.455 53.455 144.873 90 90 90	
Total reflections	50419 (4828)	
Unique reflections	6898 (665)	
Multiplicity	7.3 (7.3)	
Completeness (%)	98.34 (98.08)	
Mean I/sigma(I)	17.90 (2.79)	
Wilson B-factor	70.38	
R-merge	0.08252 (0.9043)	
R-meas	0.08902 (0.9692)	
R-pim	0.03265 (0.3441)	
CC1/2	0.997 (0.859)	
CC*	0.999 (0.961)	
Reflections used in refinement	6879 (665)	
Reflections used for R-free	688 (65)	
R-work	0.2189 (0.3054)	
R-free	0.2646 (0.3718)	
CC(work)	0.969 (0.844)	
CC(free)	0.970 (0.761)	
Number of non-hydrogen atoms	1003	
Macromolecules	1002	
Solvent	1	
Protein residues	131	
RMS(bonds)	0.010	
RMS(angles)	1.24	
Ramachandran favored (%)	96.85	
Ramachandran allowed (%)	2.36	
Ramachandran outliers (%)	0.79	
Rotamer outliers (%)	1.79	
Clashscore	5.87	
Average B-factor	76.62	
Macromolecules	76.62	
Solvent	78.27	
Statistics for the highest-resolution shell are shown in parentheses.
