# Supplementary material for: A MademoiseLLE domain binding platform links the key RNA transporter to endosomes
Source: PLoS Genet. 2022 Jun 21;18(6):e1010269. doi: 10.1371/journal.pgen.1010269 (PMC9249222; doi:10.1371/journal.pgen.1010269)
Supplement: S3 Table — (RTF) [file pgen.1010269.s013.rtf]

S3 Table: Overall SAXS Data
SAXS Device	BM29, ESRF Grenoble [1, 2]	
Data collection parameters	
Detector	PILATUS 2 M	
Detector distance (m)	2.827	
Beam size	200 µm  x 200 µm	
Wavelength (nm)	0.099	
Sample environment	Quartz capillary,1 mm ø	
s range (nm-1)‡	0.025–6.0	
Exposure time per frame (s)	1 (10 frames each concentration)	
Sample	H-Rrm4-NT4	G-Rrm4	
Organism	Ustilago maydis	Ustilago maydis	
UniProt ID and range	A0A0D1DWZ5	A0A0D1DWZ5	
Mode of measurement	Batch	Batch	
Temperature (°C)	10	10	
Protein buffer	20mM Hepes pH 8.0, 200mM NaCl, 1mM âME	20mM Hepes pH 8.0, 200mM NaCl, 1mM âME	
Protein concentration (mg/ml)	0.6	0.6	
Structural parameters	
I(0) from P(r)	41.90	103.50	
Rg (real-space from P(r)) (nm)	5.55	8.99	
I(0) from Guinier fit	43.34	104.01	
s-range for Guinier fit (nm-1)	0.060 – 0.230	0.054 – 0.147	
Rg (from Guinier fit) (nm)	5.60	8.78	
Points from Guinier fit	4 - 37	3 - 21	
Dmax (nm)	18.49	30.74	
POROD volume estimate (nm3)	122.73	586.72	
Molecular mass (kDa)	
From I(0)	43.34	104.01	
From MoW2 [3]	22.14	74.22	
From Vc [4]	36.57	147.88	
From POROD	61.37 – 76.71	293.36 – 366.70	
From sequence	40.33	110.95	
Structure Evaluation	
EOM fit ÷2	1.262	1.289	
Ambimeter score	2.307	2.530	
Software	
ATSAS Software Version [5]	3.0.3	
Primary data reduction	PRIMUS [6]	
Data processing	GNOM [7]	
Ensemble modelling	EOM [8]	
Structure evaluation	AMBIMETER [9] 	
Model visualization	PyMOL [10]	
‡s = 4ðsin(è)/ë, 2è – scattering angle, ë – X ray-wavelength
Reference
1.	Pernot, P., et al., New beamline dedicated to solution scattering from biological macromolecules at the ESRF. Journal of Physics: Conference Series, 2010. 247(1): p. 012009.
2.	Pernot, P., et al., Upgraded ESRF BM29 beamline for SAXS on macromolecules in solution. J Synchrotron Radiat, 2013. 20(Pt 4): p. 660-4.
3.	Fischer, H., et al., Determination of the molecular weight of proteins in solution from a single small-angle X-ray scattering measurement on a relative scale. Journal of Applied Crystallography, 2010. 43: p. 101-109.
4.	Rambo, R.P. and J.A. Tainer, Accurate assessment of mass, models and resolution by small-angle scattering. Nature, 2013. 496(7446): p. 477-81.
5.	Manalastas-Cantos, K., et al., ATSAS 3.0: expanded functionality and new tools for small-angle scattering data analysis. Journal of Applied Crystallography, 2021. 54(1).
6.	Konarev, P.V., et al., PRIMUS: a Windows PC-based system for small-angle scattering data analysis. Journal of Applied Crystallography, 2003. 36: p. 1277-1282.
7.	Svergun, D.I., Determination of the Regularization Parameter in Indirect-Transform Methods Using Perceptual Criteria. Journal of Applied Crystallography, 1992. 25: p. 495-503.
8.	Tria, G., et al., Advanced ensemble modelling of flexible macromolecules using X-ray solution scattering. IUCrJ, 2015. 2(Pt 2): p. 207-17.
9.	Petoukhov, M.V. and D.I. Svergun, Ambiguity assessment of small-angle scattering curves from monodisperse systems. Acta Crystallogr D Biol Crystallogr, 2015. 71(Pt 5): p. 1051-8.
10.	PyMOL, The PyMOL Molecular Graphics System, Version 2.0 Schrödinger, LLC. 2015.
