# Supplementary material for: A MademoiseLLE domain binding platform links the key RNA transporter to endosomes
Source: PLoS Genet. 2022 Jun 21;18(6):e1010269. doi: 10.1371/journal.pgen.1010269 (PMC9249222; doi:10.1371/journal.pgen.1010269)
Supplement: S4 Table — (RTF) [file pgen.1010269.s014.rtf]

S4 Table: Description of U. maydis strains used in this study
Strain name with code	Locus	Progenitor strain	Short description	
AB33
(UMa133)	b	FB2	Pnar:bW2bE1, expression of active b heterodimer under control 
of the nar1 promoter, strain grows filamentous upon changing the nitrogen source. 	
AB33rrm4/upa1-gfp
(UMa2769)	rrm4
upa1	AB33rrm4-Cherry/ upa1-gfp	carrying a deletion of rrm4 and expressing Upa1 C-terminally fused to eGfp	
AB33upa1-gfp/rrm4-kat
(UMa2976)	rrm4
upa1	AB33rrm4/upa1-gfp	expressing Upa1 C-terminally fused to eGfp and Rrm4 C-terminally fused to mKate2	
AB33upa1-gfp/rrm4-m1-kat
(UMa2977)	rrm4
upa1	AB33rrm4/upa1-gfp	expressing Upa1 C-terminally fused to eGfp and Rrm4-M1-C-terminally fused to mKate2. Like rm4-kat but carrying the deletion of 1st MLLE domain. Residues of Rrm4 from 447 to 540 were replaced with a HAtag-HRV3C protease recognition site. 	
AB33upa1-gfp/rrm4-m2-kat
(UMa2978)	rrm4
upa1	AB33rrm4/upa1-gfp	expressing Upa1 C-terminally fused to eGfp and Rrm4-M2-C-terminally fused to mKate2. Like rrm4-kat but carrying the deletion of 2nd MLLE domain. Residues of Rrm4 from 547 to 644 were replaced with a HAtag-HRV3C protease recognition site.	
AB33upa1-gfp/rrm4-m3-kat
(UMa2979)	rrm4
upa1	AB33rrm4/upa1-gfp	expressing Upa1 C-terminally fused to eGfp and Rrm4-M3-C-terminally fused to mKate2. Like rrm4-kat but carrying the deletion of 3rd MLLE domain. Residues of Rrm4 from 689 to 792 were replaced with a HAtag-HRV3C protease recognition site.	
AB33upa1-gfp/rrm4-m1,2-kat
(Uma2981)	rrm4
upa1	AB33rrm4/upa1-gfp	expressing Upa1 C-terminally fused to eGfp and Rrm4-M1,2-C-terminally fused to mKate2. Like rrm4-kat but carrying the deletion of 1st and 2nd MLLE domains. Residues of Rrm4 from 447 to 644 were replaced with a HAtag-HRV3C protease recognition site.	
AB33upa1-pl1m-gfp/rrm4-m1,2-kat
(Uma2982)	rrm4
upa1	AB33rrm4/upa1-pl1m-gfp	expressing Upa1-PL1m- C-terminally fused to eGfp, carries block mutations leading to the amino acid substitutions AASAAATAAS from residues 242-251 in the N-terminal PAM2L-motif (PAM2L-1). Rrm4-M1,2 C-terminally fused to mKate2 and carrying the deletion of 1st and 2nd MLLE domains. Residues of Rrm4 from 447 to 644 were replaced with a HAtag-HRV3C protease recognition site.	
AB33upa1-pl2m-gfp/rrm4-m1,2-kat
(Uma2983)	rrm4
upa1	AB33rrm4/upa1-pl2m-gfp
	expressing Upa1-PL2m- C-terminally fused to eGfp, carries block mutations leading to the amino acid substitutions AASAAATAAS from residues 949-958 in the C-terminal PAM2L-motif (PAM2L-2). Rrm4-M1,2 C-terminally fused to mKate2 and carrying the deletion of 1st and 2nd MLLE domains. Residues of Rrm4 from 447 to 644 were replaced with a HAtag-HRV3C protease recognition site.	
AB33upa1-pl1,2m-gfp/rrm4-m1,2-kat
(Uma3177)	rrm4
upa1	AB33rrm4/upa1-pl1,2m-gfp	expressing Upa1-PL1,2m C-terminally fused to eGfp, carries block mutations leading to the amino acid substitutions AASAAATAAS from residues 242-251 in the N-terminal PAM2L-motif (PAM2L-1) and from residues 949-958 in the C-terminal PAM2L-motif (PAM2L-2). Rrm4-M1,2 C-terminally fused to mKate2 and carrying the deletion of 1st and 2nd MLLE domains. Residues of Rrm4 from 447 to 644 were replaced with a HAtag-HRV3C protease recognition site.	
AB33upa1/rrm4-kat
(Uma3179)	rrm4
upa1	AB33upa1-gfp/rrm4-kat	Carrying a deletion of upa1 and Rrm4 C-terminally fused to mKate2	
AB33upa1-pl1,2m-gfp/rrm4-kat
(Uma3355)	rrm4
upa1	AB33rrm4/upa1-pl1,2m-gfp	expressing Upa1-PL1,2m C-terminally fused to eGfp, carries block mutations leading to the amino acid substitutions AASAAATAAS from residues 242-251 in the N-terminal PAM2L-motif (PAM2L-1) and from residues 949-958 in the C-terminal PAM2L-motif (PAM2L-2). Rrm4 C-terminally fused to mKate2.	
AB33upa1-pl1m-gfp/rrm4-kat
(UL46)	rrm4
upa1	AB33rrm4/upa1-pl1m-gfp	expressing Upa1-PL1m C-terminally fused to eGfp, carries block mutations leading to the amino acid substitutions AASAAATAAS from residues 242-251 in the N-terminal PAM2L-motif (PAM2L-1) and Rrm4 C-terminally fused to mKate2.	
AB33upa1-pl2m-gfp/rrm4-kat
(UL47)	rrm4
upa1	AB33rrm4/upa1-pl2m-gfp	expressing Upa1-PL2m C-terminally fused to eGfp, carries block mutations leading to the amino acid substitutions AASAAATAAS from residues 949-958 in the C-terminal PAM2L-motif (PAM2L-2) and Rrm4 C-terminally fused to mKate2.	
AB33upa1-pl2m-gfp/rrm4-m1-kat 
(UL48)	rrm4
upa1	AB33rrm4/upa1-pl1,2m-gfp	expressing Upa1-PL2m C-terminally fused to eGfp, carries block mutations leading to the amino acid substitutions AASAAATAAS from residues 949-958 in the C-terminal PAM2L-motif (PAM2L-2). Rrm4-M1 C-terminally fused to mKate2. Like rrm4-kat but carrying the deletion of 1st MLLE  domain. Residues of Rrm4 from 447 to 540 were replaced with a HAtag-HRV3C protease recognition site.	
AB33upa1-pl2m-gfp/rrm4-m1,2-kat
(UL49)	rrm4
upa1	AB33rrm4/upa1-pl2m-gfp	expressing Upa1-PL2m C-terminally fused to eGfp,  carries block mutations leading to the amino acid substitutions AASAAATAAS from residues 949-958 in the C-terminal PAM2L-motif (PAM2L-2). Rrm4-M1,2 C-terminally fused to mKate2 and carrying the deletion of 1st and 2nd MLLE domains. Residues of Rrm4 from 447 to 644 were replaced with a HAtag-HRV3C protease recognition site.	
