# Supplementary material for: A MademoiseLLE domain binding platform links the key RNA transporter to endosomes
Source: PLoS Genet. 2022 Jun 21;18(6):e1010269. doi: 10.1371/journal.pgen.1010269 (PMC9249222; doi:10.1371/journal.pgen.1010269)
Supplement: S5 Table — (RTF) [file pgen.1010269.s015.rtf]

S5 Table: Generation of U. maydis strains used in this study
Strains	Relevant genotype	Strain code	Reference	Transformed
plasmid	Locus	Progenitor	
AB33	a2 Pnar:bW2 bE1	UMa      133	[1]	pAB33	b	FB2	
AB33rrm4/upa1-gfp	rrm4
upa1-gfp	UMa 2769	this study	pRrm4_genitR
(pUMa1755)	rrm4
	AB33rrm4-mCherry/upa1-gfp
(UMa1594)	
AB33upa1-gfp/rrm4-kat	upa1-gfp
rrm4-kat	Uma 2976	this study	pRrm4-kat-hygR
(pUMa3908)	rrm4	AB33rrm4/upa1-gfp
(UMa2769)	
AB33upa1-gfp/rrm4-m1-kat	upa1-gfp 
rrm4-m1-kat	UMa 2977	this study	pRrm4-m1-kat-hygR
 (pUMa4433)	rrm4	AB33rrm4/upa1-gfp
(UMa2769)	
AB33upa1-gfp/rrm4-m2-kat	upa1-gfp
rrm4-m2-kat	UMa 2978	this study	pRrm4- m2-kat-hygR
(pUMa4434)	rrm4	AB33rrm4/upa1-gfp
(UMa2769)	
AB33upa1-gfp/rrm4-m3-kat	upa1-gfp
rrm4-m3-kat	UMa 2979	this study	pRrm4-m3-kat-hygR
 (pUMa4435)	rrm4	AB33rrm4/upa1-gfp
(UMa2769)	
AB33upa1-gfp/rrm4-m1,2-kat 	upa1-gfp
rrm4-m1,2-kat	UMa 2981	this study
	pRrm4-m1,2-kat-hygR
(pUMa4578)	rrm4	AB33rrm4/upa1-gfp
(UMa2769)	
AB33upa1-pl1m-gfp/
rrm4- m1,2-kat	upa1-pl1m-gfp rrm4-m1,2-kat	UMa 2982	this study	pRrm4-m1,2-kat-hygR
 (pUMa4578)	rrm4	AB33rrm4/upa1-pl1m-gfp
(UMa2766)	
AB33upa1-pl2m-gfp/
rrm4-m1,2-kat	upa1-pl2m-gfp rrm4-m1,2-kat	UMa 2983	this study	pRrm4-m1,2-kat-hygR
(pUMa4578)	rrm4	AB33rrm4/upa1-pl2m-gfp
(UMa2767)	
AB33upa1-pl1,2m-gfp/
rrm4-m1,2-kat	upa1-pl1,2m-gfprrm4-m1,2-kat	UMa 3177	this study	pRrm4-m1,2-kat-hygR
(pUMa4578)	rrm4	AB33rrm4/upa1-pl1,2m-gfp
(UMa2768)	
AB33upa1/rrm4-kat	upa1
rrm4-kat	UMa 3179	this study	pUpa1-genitR
(pUMa1915)	upa1
	AB33upa1-gfp/rrm4-kat 
(UMa2976)	
AB33upa1-pl1,2m-gfp/
rrm4-kat	upa1-pl1,2m-gfp
rrm4-kat	UMa 3355	this study
	pRrm4-kat-hygR
(pUMa3908)	rrm4	AB33rrm4/upa1-pl1,2m-gfp
 (UMa2768)	
AB33upa1-pl1m- gfp/
rrm4-kat	upa1-pl1m- gfp
rrm4-kat	UL46	this study	pRrm4-kat-hygR
(pUMa3908)	rrm4	AB33rrm4/upa1-pl1m-gfp
 (UMa2766)	
AB33upa1-pl2m-gfp/
rrm4-kat	upa1-pl2m- gfprrm4-kat	UL47	this study	pRrm4-kat-hygR
(pUMa3908)	rrm4	AB33rrm4/upa1-pl2m-gfp
 (UMa2767)	
AB33upa1-pl2m-gfp/
rrm4-m1-kat	upa1-pl2m- gfp 
rrm4-m1-kat	UL48	this study	pRrm4-m1-kat-hygR
(pUMa4433)	rrm4	AB33rrm4/upa1-pl2m-gfp
 (UMa2767)	
AB33upa1-pl2m-gfp/
rrm4-m2-kat	upa1-pl2m- gfp
rrm4-m2-kat	UL49	this study	pRrm4-m2-kat-hygR
(pUMa4434)	rrm4	AB33rrm4/upa1-pl2m-gfp
 (UMa2767)	

Reference
1.	Brachmann A, Weinzierl G, Kämper J, Kahmann R. Identification of genes in the bW/bE regulatory cascade in Ustilago maydis. Mol Microbiol. 2001 42:1047-63. https://doi.org/10.1046/j.1365-2958.2001.02699.x. PMID: 11737646.
