# Supplementary material for: A MademoiseLLE domain binding platform links the key RNA transporter to endosomes
Source: PLoS Genet. 2022 Jun 21;18(6):e1010269. doi: 10.1371/journal.pgen.1010269 (PMC9249222; doi:10.1371/journal.pgen.1010269)
Supplement: S6 Table — (RTF) [file pgen.1010269.s016.rtf]

S6 Table: Description of plasmids used for U.  maydis strain generation
Plasmid	pUMa	Resistance cassette	Short description	
pRrm4	1755	genitR (G418 resistance - SfiI insert of pMF1g) [1] 	Plasmid vector for generating deletion mutants of rrm4. 	
pUpa1_genitR	1915	genitR [2]	Plasmid vector for generating deletion mutants of upa1.  	
pRrm4-kat-hygR	3908	hygR (Hygromycin resistance - SfiI insert 
of pMF1h) [3]	Plasmid vector for the expression of Rrm4 C-terminally fused to mKate2. The mKate2 cassette contains the Tnos terminator and the Hyg resistance. The entire coding sequence for the fusion protein is flanked by a 1025 bp upstream region and a 1396 bp downstream region for homologous recombination. 	
pRrm4-m1-kat-hygR	4433	hygR 	Plasmid vector for the expression of Rrm4-M1  C-terminally fused to mKate2. Like pRrm4-mK-HygR, but carrying the deletion of 1st MLLE domain. Residues of Rrm4 from 447 to 540 were replaced with a HAtag-HRV3C protease recognition site.	
pRrm4-m2-kat-hygR	4434	hygR	Plasmid vector for the expression of Rrm4-M2  C-terminally fused to mKate2. Like pRrm4-mK-HygR, but carrying the deletion of the 2nd MLLE domain. Residues of Rrm4 from 547 to 644 were replaced with a HAtag-HRV3C protease recognition site.	
pRrm4-m3-kat-hygR	4435	hygR	Plasmid vector for the expression of Rrm4-M3 C-terminally fused to mKate2. Like pRrm4-mK-HygR, but carrying the deletion of the 3rd MLLE domain. Residues of Rrm4 from 689-792 were replaced with a HAtag-HRV3C protease recognition site.	
pRrm4-m1,2-kat-hygR	4578	hygR	Plasmid vector for the expression of Rrm4-M1,2 C-terminally fused to mKate2. Like pRrm4-mK-HygR, but carrying the deletion of 1st and 2nd MLLE domains. Residues of Rrm4 from 447 to 644 were replaced with a HAtag-HRV3C protease recognition site.	

1.	Baumann S, Pohlmann T, Jungbluth M, Brachmann A, Feldbrügge M. Kinesin-3 and dynein mediate microtubule-dependent co-transport of mRNPs and endosomes. J Cell Sci. 2012; 125:2740-52. https://doi.org/10.1242/jcs.101212 PMID: 22357951
2.	Pohlmann T, Baumann S, Haag C, Albrecht M, Feldbrügge M. A FYVE zinc finger domain protein specifically links mRNA transport to endosome trafficking. Elife. 2015 4:e06041. https://doi.org/10.7554/eLife.06041. PMID: 25985087
3.	Brachmann A, König J, Julius C, Feldbrügge M. A reverse genetic approach for generating gene replacement mutants in Ustilago maydis. Mol Genet Genomics. 2004 272:216-26. https://doi.org/10.1007/s00438-004-1047-z. PMID: 15316769
