# Supplementary material for: A MademoiseLLE domain binding platform links the key RNA transporter to endosomes
Source: PLoS Genet. 2022 Jun 21;18(6):e1010269. doi: 10.1371/journal.pgen.1010269 (PMC9249222; doi:10.1371/journal.pgen.1010269)
Supplement: S7 Table — (RTF) [file pgen.1010269.s017.rtf]

S7 Table: Description of plasmids used for recombinant expression in E. coli
Plasmid	pUMa	Short description	
pGEX-G-Pab1-MLLE	2187	Plasmid for the expression of the G-Pab1-MLLE. C terminal region of Pab1 comprising amino acid residues 566-651 were N-terminally fused to a GST-tag. [1]	
pGEX-G-Rrm4-NT4	3920	Plasmid for the expression of the G-Rrm4-NT4. C terminal region of Rrm4 comprising amino acid residues 421 to 792 was N-terminally fused to a GST-tag.	
pGEX-G-Rrm4-NT4-M1	4616	Plasmid for the expression of the G-Rrm4-NT4-M1. Same as pUMa3920 but carrying the deletion of 1st MLLE domain. Residues of Rrm4 from 447 to 540 were replaced with a HAtag-HRV3C protease recognition site.	
pGEX-G-Rrm4-NT4-M2	4617	Plasmid for the expression of the G-Rrm4-NT4-M2. Same as pUMa3920 but carrying the deletion of the 2nd MLLE domain. Residues of Rrm4 from 547 to 644 were replaced with a HAtag-HRV3C protease recognition site.	
pGEX-G-Rrm4-NT4-M3	4618	Plasmid for the expression of the G-Rrm4-NT4-M3. Same as pUMa3920 but carrying the deletion of the 3rd MLLE domain. Residues of Rrm4 from 689 to 792 were replaced with a HAtag-HRV3C protease recognition site.	
pGEX-G-Rrm4-NT4-M1,2	4619	Plasmid for the expression of the G-Rrm4-NT4-M1,2. Same as pUMa3920 but carrying the deletion of 1st to 2nd MLLE domains. Residues of Rrm4 from 447 to 644 were replaced with a HAtag-HRV3C protease recognition site.	
pET28-HS-PAM2Upa1	4296	Plasmid for the expression of the PAM2 motif of Upa1 (SQSTLSPNASVFKPSRS) as a fusion protein with an N terminal 6xHis-Sumo-tag.	
pET28-HS_PAM2L1Upa1	4297	Plasmid for the expression of PAM2L1 motif of Upa1 (EAADQEEDQDDFVYPGAD) as a fusion protein with an N terminal 6xHis-Sumo-tag.	
pET28-HS-PAM2L2Upa1	4298	Plasmid for the expression of PAM2L2 motif of Upa1 (DEDAADDDDDEFIYPNSY) as a fusion protein with an N terminal 6xHis-Sumo-tag.	
pET22-H-Rrm4-NT4	3552	Plasmid for the expression of H-Rrm4-NT4. C terminal region of Rrm4 comprising amino acid 421 to 792 were N-terminally fused to 6xHis-tag.	
pGX-G-Rrm4	429	Plasmid for the expression of G-Rrm4. Rrm4 full-length protein was N-terminally fused to GST.	

Reference
1.	Pohlmann T, Baumann S, Haag C, Albrecht M, Feldbrügge M. A FYVE zinc finger domain protein specifically links mRNA transport to endosome trafficking. Elife. 2015 4:e06041. https//:10.7554/eLife.06041. PMID: 25985087
