# Supplementary material for: A MademoiseLLE domain binding platform links the key RNA transporter to endosomes
Source: PLoS Genet. 2022 Jun 21;18(6):e1010269. doi: 10.1371/journal.pgen.1010269 (PMC9249222; doi:10.1371/journal.pgen.1010269)
Supplement: S8 Table — (RTF) [file pgen.1010269.s018.rtf]

S8 Table: Description of plasmids used for yeast two-hybrid analyses
Plasmid	Plasmid code	Gene	Short description	
pGADT7-DS 	pUMa1624		Plasmid for the expression of hybrid proteins, N-terminally fused to a nuclear localisation signal (NLS) of the simian virus 40 (SV40), followed by the Gal4 activation domain (aa 768-881) and an HA-epitope for Western Blot detection. The resulting hybrid proteins are termed AD-“X”. For the positive selection of transformants on minimal medium, this plasmid carries a LEU2 auxotrophy marker. This plasmid contains two diverse SfiI-restriction sites for cloning purposes (Dualsystems Biotech, Schlieren, Switzerland).	
pGBKT7-SfiI MCS 	pUMa1625		Plasmid for the expression of hybrid proteins, N-terminally fused to the Gal4 DNA-binding domain (aa 1-147), followed by a c-Myc-epitope for Western Blot detection. The resulting hybrid proteins are termed BD-“X”. For the positive selection of transformants on minimal medium, this plasmid carries a TRP1 auxotrophy marker. This plasmid contains two diverse SfiI-restriction sites for cloning purposes. (Clontech Laboratories, Inc., Mountain View, CA, USA).	
pGADT7-T 	pUMa1636		Plasmid for the expression of an N-terminal AD-fusion of the large T-antigen of SV40. It interacts with BD-p53 as a positive control (Clontech).	
pGBKT7-p53 	pUMa1638		Plasmid for the expression of an N-terminal BD-fusion of the murine p53. It interacts with AD-T as a positive control (Clontech).	
pGBKT7-Lam 	pUMa1637		Plasmid for the expression of an N-terminal BD-fusion with the human nuclear protein Lamin C, which shows no interaction with most proteins and serves as negative control (Clontech).	
pGBKT7-Upa1-Gfp 
	pUL0128	upa1
	Plasmid for the expression of BD-Upa1-Gfp, where eGfp is fused C-terminally to the BD-Upa1-hybrid.	
pGBKT7-Upa1-pl1m-Gfp	pUL0120	upa1	Like GBKT7-Upa1-Gfp, expressing BD-Upa1-pl1-Gfp, where eGfp is fused C-terminally to the BD-Upa1-pl1m hybrid but carries block mutations leading to the amino acid substitutions AASAAATAAS from residues 242-251 in the N-terminal PAM2L-motif (PAM2L-1) of Upa1.	
pGBKT7-Upa1-pl2m-Gfp	pUL0121	upa1	Like pGBKT7-Upa1-Gfp, expressing BD-Upa1-pl2-Gfp, where eGfp is fused C-terminally to the BD-Upa1-pl2m hybrid. but carries block mutations leading to the amino acid substitutions AASAAATAAS from residues 949-958 in the C-terminal PAM2L-motif  (PAM2L-2) of Upa1.	
pGBKT7-Upa1-pl1,2m-Gfp	pUL0122	upa1	Like GBKT7-Upa1-Gfp, expressing BD-Upa1-pl1,2-Gfp, where eGfp is fused C-terminally to the BD-Upa1-pl1,2m hybrid but carries block mutations leading to the amino acid substitutions AASAAATAAS from residues 242-251 in the N-terminal PAM2L-motif (PAM2L-1) and from residues 949-958 in the C-terminal PAM2L-motif (PAM2L-2).	
pGADT7-Rrm4-kat	pUL0112	rrm4	Plasmid for the expression of AD-Rrm4-kat, where mKate2 is fused C-terminally to the AD-Rrm4-hybrid.	
pGADT7-Rrm4-m1-kat	pUL0116	rrm4	Plasmid for the expression of AD-Rrm4-M1-kat  where mKate2 is fused C-terminally to the AD-Rrm4-M1 hybrid. Like pGADT7-Rrm4-kat, but carrying the deletion of 1st MLLE domain. Residues of Rrm4 from 447 to 540 were replaced with a HAtag-HRV3  C protease recognition site.	
pGADT7-Rrm4-m2-kat	pUL0117	rrm4	Plasmid for the expression of AD-Rrm4-M2-kat, where mKate2 is fused C-terminally to the AD-Rrm4-M2 hybrid. Like pGADT7-Rrm4-kat, but carrying the deletion of 2nd MLLE domain. Residues of Rrm4 from 547 to 644 were replaced with a HAtag-HRV3C protease recognition site.	
pGADT7-Rrm4-m1,2-kat	pUL0118	rrm4	Plasmid for the expression of AD-Rrm4-M1,2-kat, where mKate2 is fused C-terminally to the AD-Rrm4-M1,2 hybrid. Like pGADT7-Rrm4-kat, but carrying the deletion of 1st and 2nd MLLE domains. Residues of Rrm4 from 447 to 644 were replaced with a HAtag-HRV3C protease recognition site. 	
pGADT7-Rrm4-m3-k	pUL0119	rrm4	Plasmid for the expression of AD-Rrm4-M3-kat, where mKate2 is fused C-terminally to the AD-Rrm4-M3 hybrid. Like pGADT7-Rrm4-kat, but carrying the deletion of 3rd MLLE domain. Residues of Rrm4 from 689-792 were replaced with a HAtag-HRV3C protease recognition site.	
