# Supplementary material for: A MademoiseLLE domain binding platform links the key RNA transporter to endosomes
Source: PLoS Genet. 2022 Jun 21;18(6):e1010269. doi: 10.1371/journal.pgen.1010269 (PMC9249222; doi:10.1371/journal.pgen.1010269)
Supplement: S9 Table — (RTF) [file pgen.1010269.s019.rtf]

S9 Table: DNA oligonucleotides used in this study
Designation	Nucleotide sequence (5' --> 3')	Remarks	
oUM727	GTATTCGAGCCAAGCATCTACGTATGTCGACCCTTGCAACC	Rrm4-internal-Gibson cloning-fwd	
oAB354	GGGCCCCTGGAACAGTACTTCCAGGGCGTAGTCGGGCACGTCGTAAGGGTAAGGCACACCTGCTTTGAAG	Rrm4-M1_Gibson cloning-rev	
oAB355	TACCCTTACGACGTGCCCGACTACGCCCTGGAAGTACTGTTCCAGGGGCCCCTGTCTGCTGAACACCCAGC	Rrm4-M1_Gibson cloning-fwd	
oAB359	CGATCGCCGGGCGGCCGGCGCGCCACCGGTTTAGCGGTGACCGAGTTTCGAGG	mKate2-rev	
oAB345	GGGCCCCTGGAACAGTACTTCCAGGGCGTAGTCGGGCACGTCGTAAGGGTATGCAGGAAGCGCAGCAAGCG	Rrm4-M3_Gibson cloning-rev	
oAB346	TACCCTTACGACGTGCCCGACTACGCCCTGGAAGTACTGTTCCAGGGGCCCGCGGCCAACGCGGCCACCATGGTG	Rrm4-M3_Gibson cloning-fwd	
oAB356	GGGCCCCTGGAACAGTACTTCCAGGGCGTAGTCGGGCACGTCGTAAGGGTATGGGTGTTCAGCAGACAGTG	Rrm4-M2_Gibson cloning-rev	
oAB357	TACCCTTACGACGTGCCCGACTACGCCCTGGAAGTACTGTTCCAGGGGCCCAGCGCTCCGGTGCCATTGTC	Rrm4-M2-Gibson cloning-fwd	
oAB312	CATGCCATGGCCAGCAGCAACAGTCCGCCCAC	NcoI_Rrm4-NT4-fwd	
oAB45	CGGCCATATGGGCAGCAGCCATCATC	pET28 vector-ORF-fwd	
oAB46	CTCACTCGAGTTAGGATCGGGACGGCTTGAAGACGGAGGCGTTGGGAGACAAGGTGCTTTGCGAACCACCAATCTGTTCTCTGTGAGC	Sumo-PAM2-XhoI-rev	
oAB47	CTCACTCGAGTTAGTCGGCTCCTGGGTAGACAAAGTCATCTTGATCTTCCTCTTGGTCTGCAGCCTCACCACCAATCTGTTCTCTGTGAG	Sumo-PAM2L1-XhoI-rev	
oAB48	CTCACTCGAGTTAGTACGAGTTCGGGTAGATGAATTCATCGTCATCGTCATCGGCCGCATCCTCGTCACCACCAATCTGTTCTCTGTGAG	Sumo-PAM2L2-XhoI-rev	
oMB696	TCGACTCGAGTCACTTGTTCAGACCTGCAGC	Rrm4- XhoI-rev	
oUP605	GGGAATTCCATATGCATCATCATCATCATCACAGCAGCAACAGTCCGCCCAC	NdeI-6xHis-Rrm4-NT4 fwd	
