# Supplementary material for: A MademoiseLLE domain binding platform links the key RNA transporter to endosomes
Source: PLoS Genet. 2022 Jun 21;18(6):e1010269. doi: 10.1371/journal.pgen.1010269 (PMC9249222; doi:10.1371/journal.pgen.1010269)
Supplement: Attachment — Submitted filename: 121591_1_rebuttal_2323592_r99c5c.pdf [file pgen.1010269.s021.pdf]

Full Revision

Manuscript number: RC-2021-01123
Corresponding author(s): Sander HJ Smits, Holger Gohlke and Michael Feldbrügge

1. General Statements

Endosomal transport of mRNAs is an evolutionarily conserved trafficking mechanism important in higher plants, fungi and neurons. Numerous components have been identified, but little is known about the precise mechanisms of attachment. Structural and mechanistic insights are necessary to understand this trafficking process.

We are very grateful for the constructive criticism that we received, and in particular, the cross comments are highly valuable. A significant point of criticism was that we did not show conclusively that MLLE2 indeed has a function in the attachment of endosomes shuttling along microtubules. In our improved version, we now quantified from kymographs that Rrm4 with mutations in MLLE2 exhibits reduced intensity of signals showing processive movement. Thus, if MLLE2 is mutated, the linkage of Rrm4 to endosomes is clearly affected, and mistargeted proteins result in aberrant staining of microtubules. We included important additional control experiments demonstrating that (i) aberrant staining is abolished in the presence of benomyl, a drug that inhibits the polymerisation of microtubules. Hence, the mislocalisation of Rrm4 is microtubule-dependent (ii) mutations to do not affect amounts of Rrm4 variants and the interactions of Rrm4 and adaptor protein Upa1 can be recapitulated testing full-length proteins.

Thus, we firmly believe that the improved version of the manuscript substantially advances our knowledge of RNA trafficking. This publication describes the currently most advanced system clarifying how mRNPs are linked to the cytoplasmic surface of transport endosomes by the interaction of the key mRNA transport protein via a sophisticated binding platform. Importantly, this novel binding platform works with a strict hierarchy: MLLE3 is the main domain, MLLE2 plays an accessory function during endosomal attachment, and MLLE1 has a yet unknown function.

The description of a tripartite MLLE binding platform is unprecedented, but the underlying principle of main and accessory domains is most likely conserved in other systems. Our findings are not only crucial for the endosomal transport but also shed new light on the function of MLLE domains in general. The importance is highlighted by the poly(A)-binding protein Pab1 interacts with all mRNAs in the cell using the described domain to carry out essential functions in RNA biology.

In essence, our results are of interest for a broad readership interested in microbiology, RNA biology, membrane trafficking, and readers interested in structural biology. In the latter case, we demonstrate the successful interplay of X-ray crystallography, SAXS and molecular modelling. Thus, we believe that our improved manuscript is well-suited for publication in your journal.

The project is a tight collaboration of structural modellers, biochemists and fungal cell biologists. Therefore, Dr. Gohlke, Dr. Smits and I would like to serve as shared co-corresponding authors.

Full Revision

Please find below our response to the reviewer comments printed in bold.

2. Point-by-point description of the revisions

Reviewer #1 (Evidence, reproducibility and clarity (Required)):

Summary: This manuscript documents a very thorough biophysical, structural and functional dissection of interactions between the RNA-binding protein Rrm4 and the endosomal adaptor Upa1 in the filamentous fungus Ustilago maydis. It has been shown previously that the Rrm4-Upa1 interaction is critical for mRNA transport in this system as mRNAs hitchhike on motor-associated endosomes. Here, the authors reveal using modelling that Rrm4 has three MLLE domains, including a cryptic one that had not been identified previously. They then report the crystal structure of MLLE2 and analyze the distribution anf arrangement of the MLLE domains in the protein using SAXS. They then show using pulldowns and isothermal titration calorimetry that MLLE3 is critical for the Upa1 interaction (via the PAM2L domains of Upa1) and that MLLE2 contributes to Rrm4 localization in vivo when the MLLE3-Upa1 interaction is partially impaired. The study suggests that Rrm4 has a platform of MLLE domains for orchestrating Rrm4 function. Overall, this is technically a high quality study. However, a number of points (mostly minor) should be addressed.

Major comments:

A key part of the study if the in vivo work illustrating a role for MLLE2 in regulating Rrm4 localization when the system is sensitized. Some aspects of this part of the work need clarifying.

a) The authors should show that the abberant staining is indeed microtubule-related with the benomyl experiment that they used in Jankowski et al. 2019.
We included this important control in Figure EV5F demonstrating that the aberrant staining is no longer visible after the microtubule inhibitor benomyl treatment.

b) The authors claim from these experiments that MLLE2 contributes to endosomal targeting (as there is ectopic protein on other structures (presumptive microtubules)). However, to make this claim, the authors would need to measure the intensity of the mutant Rrm4 protein on endosomes and/or the colocalization of these Rrm4 variants with endosomes, as they do in other experiments in this paper. Otherwise, it is possible that the MLLE2 deletion has another effect, e.g. increasing protein stability, and thus increasing the likelihood of binding to structures other than endosomes. If available, data on the

relative abundance in the cell of the protein expressed from the wild-type control (rrm4-kat) and MLLE2 deletion constructs (e.g. rrm4-m1,2delta-kat) should be provided.
As indicated by the reviewer, a critical point is identifying a function of MLLE2. Surprisingly, the domain is conserved in evolution, but , we do not see a mutant phenotype under optimal culture conditions. Therefore, we challenged the system and observed the mislocalisation of Rrm4, if the MLLE2 domain is deleted. However, the overall amount of shuttling Rrm4-positive endosomes was not strongly affected according to our kymograph experiments. We observe aberrant staining, which is not seen with the Rrm4 wild-type protein. Thus, under challenging conditions, we do see a function of MLLE2.
To address the valid point of the reviewer, we quantified the signal intensities in kymographs of the most important Rrm4 variants. As indicated in Figure 5E, we observed that the maximum fluorescence intensity in kymograph signals was reduced when Rrm4 variants are mislocalised to microtubules while the minimum intensities were comparable in all strains. This underlines that a subset of Rrm4 molecules are no longer shuttling through the cell and most likely are attached to microtubules (to prove the involvement of microtubules, we did benomyl treatment which is now shown in Figure EV5F). We also included a Western Blot experiment (Figure EV5G) demonstrating that neither MLLE1 nor MLLE2 deletion impacts the total protein amount of Rrm4. These data support the notion that MLLE2 contributes to endosomal targeting.

c) Was the data in Figure 5D scored blind of the identity of the samples? Given that the classification has to be done manually, it is important to confirm the phenotypes are robust to blinding (at least for the key comparisons).
We agree entirely that manual evaluation of microscopic images has to be carried out with utmost care. The phenotype of aberrant microtubule staining is not easily detectable, and it needs an experienced person to quantify this. The data were analyzed by a second experimentalist with experience in evaluating microscopy images to validate the system's robustness. Notably, the key findings were confirmed in both cases aberrant microtubule staining was only observed when the MLLE domain was mutated. However, the second person reported difficulties in differentiating a bundle of Rrm4 signals or stained microtubules. Therefore, this person quantified higher values with less experience in Rrm4 movement. In essence, we can rely on the key findings. We included the information in the section "Materials and methods" and gave the comparison in Figure EV5H.

If points b and c are addressed, it should be possible to draw an arrow between the gray question mark protein in Figure 6 and the endosome surface, which is what I assume the authors believe to be case based on their discussion.
Having addressed both points, we have also improved the model. To this end, we added a second unknown protein component (grey oval with a question mark) that interacts with MLLE2 and the endosomal surface. Thereby the hierarchical order with the accessory role of MLLE2 during endosomal attachment is stressed.

Minor comments:

1. The first line of the abstract is quite bold. It is hard to quantify the role of transport vs RNA stability for example, so I suggest this sentence is toned down.
Correct, the first line now reads, “Spatiotemporal expression can be achieved by transport and translation of mRNAs at defined subcellular sites”.

2. Line 269: change "amount of motile Rrm4-M12delta-Kat positive signals" to "number of motile Rrm4-M12delta-Kat positive signals".
Changed as mentioned above.

3. Figure 3 legend: Insert "Variant" before "amino acids of the FxP and FxxP..." to indicate what is labeled in gray. Change "fond" to "font" in the same sentence.
Corrected as mentioned above.

4. The cartoons of the different protein variants are very helpful but I had problems spotting the Upa1-Pam2L deletions due to the similar gray to the background of the protein. This would perhaps be clearer if the gray used for the background was lighter than it currently is.
We improved the contrast by reducing the background of Upa1 to a lighter grey tone in all the corresponding figures.

5. The residual motility of wild-type Rrm4 when PAM2L1 and PAM2L2 are both mutated (Figure 5C) is reminiscent of what is seen in a complete Upa1 deletion in the group's previous work. It would be helpful to point this out to the reader, as well as the implication that other proteins are contributing to Rrm4's linkage to endosomes. After all, some of these other adaptors might contact MLLE2 of Rrm4.
We addressed this point by referring to our previous publication with the following sentence: “Comparable to previous reports, we observed residual motility of Rrm4-Kat on shuttling the endosomes if both PAM2L motifs are mutated or if *upa1* is deleted. This indicates that additional proteins besides Upa1 are involved in the endosomal attachment of Rrm4 (Pohlmann *et al.*, 2015).”

6. Some of the y-axes of the charts should be more descriptive so that the reader can understand the plots even before they consult the legends. For example, in Figure EV4A and EV5D and E, which protein is being to referred to in each 'number of signals' plot should be included. In Figure 5D, 'Hyphae [%]' would be clearer as 'Hyphae with MT staining of Rrm4 [%]'
We improved this in Figures EV4, 5D and EV5.

7. Figure EV5 legend title: this could be misleading as the authors are seeing ectopic MT localization rather than a deficit in microtubule association.

Corrected to "Deletion of MLLE1^{Rrm4} and -2 cause aberrant staining of microtubules".

Reviewer #1 (Significance (Required)):
The Feldbrugge group has previously mapped interactions between Upa1 and Rrm4 (Pohlmann et al., 2015) and some conclusions are corroborated in the paper by Boehm et al. The paper under review is, however, a significant advance due to the identification of the third MLLE domain, detailed biophysical characterization of the interactions, the structural insights, and evidence of a subsidiary role of MLLE2. The work would of course be stronger if the target of MLLE2 had been identified but I think this is beyond the scope of this initial work. To my knowledge, this is one of the most extensive analyses of the interactions mediated by MLLE and PAM domains and will be of interest to others working on these protein features. The work will also appeal to those interested in the links of localizing mRNAs with motor-associated membranes, which is an emerging field.

Reviewer expertise: I have a long-standing interest in molecular analysis of mRNA trafficking mechanisms. I do not have experience in fungal genetics.

****Referee Cross-commenting****
It seems that we are in agreement that this is solid work and that biochemical and biophysical analysis of the MLLE-PAM interactions will be of significant interest to those working on those domains (or proteins containing those domains). I agree with the comments of the other reviewers and there are clearly some essential minor revisions needed to strengthen the evidence for their conclusions and some clarifications. I think it is a long shot that RNA binding to the RRMs will affect the MLLE-PAM interactions and would require quite a lot of work to show this conclusively. The study would, however, be more impactful if this was shown to be the case, or the target of MLLE2 was found. Nonetheless, I would not say these new avenues of research are necessary to find a home in one of the Review Commons journals.

Reviewer #2 (Evidence, reproducibility and clarity (Required)):
Devan, Schott-Verdugo et al.
Summary
In this study the putative MLLE RNA-binding motifs of the endosomal RNA-binding protein, Rrm4, from Ustilago maydis were examined using structural and genetic analyses. MLLE motifs are conserved in polyA-binding proteins (Pab1/PABPC1) and found also in Rrm4, which was shown to reside on motile endosomes and deliver septin mRNAs for endosome-localized translation during polarized growth. Upa1 on the endosome interacts with Rrm4 via its PAM2L domain that itself interacts with the MLLE domains of proteins like Pab1. Mutations in the known MLLE domain of Rrm4 were earlier shown to affect localization to endosomes.
Here, the C-terminal domain of Rrm4 was revealed to have three divergent MLLE motifs using comparative modeling; only two of which were previously predicted. Crystallization and X-ray diffraction analysis of a truncated version of bacterially produced Rrm4, showed

MLLE2 is most similar to that of PABPC1 and UBR5, although MLLE1 and 2 are somewhat divergent in the key region of PAM2 binding. Small angle X-ray scattering of recombinant full-length or truncated Rrm4 revealed that the MLLE domains might form a platform that could allow for multiple contacts with different binding partners. In vitro binding studies with different N-terminal GST-tagged versions of the Rrm4 were used to examine for interactions with PAM2 sequences of Upa1 using N-terminal hexa-histidine-SUMO fusions. It was found that Pab1-MLLE interacts with the PAM2, but not PAM2L, domain of Upa1. In contrast, the complete Rrm4 MLLE region (G-Rrm4-NT4) interacted with the PAM2L domain, but not the PAM2 of Upa1. Notably, the interaction with PAM2L required the third MLLE and neither MLLE1 nor MLLE2, nor both. No significant differences in affinity were observed and were similar to that of the Pab1 MLLE. The results also show that the MLLE3 has a higher affinity for the PAM2L2 than PAM2L1 of Upa1.
To examine the biological role of the Rrm4 MLLEs, U. maydis strains bearing deletions in the domains of Rrm4 were examined for hyphal growth and endosomal transport (latter using Upa1-GFP and Rrm4-mKate2). Only the loss of the MLLE3 domain inhibited polarized growth (as seen with the full deletion of RRM4) and not the deletion of either MLLE1 or 2. Similar results were obtained regarding endosome shuttling. Thus, in line with the biochemical experiments performed the MLLE3 domain alone (of the three identified) is necessary for the biological actions of Rrm4. This suggested the MLLE1 and 2 are not necessary for function under these conditions.
To examine this further, Upa1 carrying mutations in the PAM2L 1or PAM2L2 domains were examined. It was found that the deletion of both PAM2L domains affected unipolar growth resulting in bipolar growth similar to the deletion of UPA1 alone. This phenotype was observed even upon the deletion of Rrm4 MLLE1 and 2 in the same background as the PAM2L mutants. The mutation of both PAM2L domains led to a reduction in Rrm4-labeled shuttling endosomes, which suggests that these domains help anchor Rrm4 to endosomes. When only the PAM2L1 domain is present in Upa1 there was a larger increase in hyphae with aberrant microtubule staining than upon the loss of PAM2L1. The authors suggest that this indicates PAM2L2 is more important and prescribes an accessory role for MLLE2 in endosome association.

Comments:
Overall, the study seems well conducted. We cannot comment on the structural aspect of the work since this is not our field of expertise. That said, the biochemical and genetic/functional studies appear solid, well thought-out, and clearly presented. No new experiments are necessary to support the general claims of the paper, however, experiments suggested below might make it more revealing with regards to the connection between RNA binding and MLLE-PAM2L interactions (i.e. endosome localization and RNA binding functions).

1. Line 286 - It reads the they "Next, we investigated the association of Rrm4 -M12D-Kat in strains expressing PAM2L1. Thus, the endosomal attachment was solely dependent on the

interaction of MLLE3 with the PAM2L2 sequence of Upa1." Unclear - wouldn't lacking PAM2L1 (and not expressing) fit the logic of the sentence?
We corrected this with the sentence, “Next, we investigated the association of Rrm4-M1,2Δ-Kat in strains expressing Upa1 with mutated PAM2L1”.

2. Several questions regarding the specificity of PAM2 vs. PAM2L domains. What happens when you switch/replace the PAM2L1 or 2 of Upa1 with Upa1 PAM2 domains? Are they exclusive? What happens when the MLLE3 of Rrm4 is switched with that of Pab1? And if one does both - does that restore functionality to Rrm4?
These are very interesting suggestions. Previously, we have shown that a single PAM2L1 or PAM2L2 sequence of Upa1 is sufficient for unipolar growth and recruitment of Rrm4 to endosomes. Please note that Upa1 with mutated PAM2L1 and L2 still contains a PAM2 motif. Furthermore, mutating the PAM2 motif of Upa1 did not affect Rrm4 shuttling or unipolar growth. Thus, switching the domains would mostly address whether the precise location within Upa1 would be important. This is interesting but, unfortunately very labour-intensive and beyond the manuscript’s current scope.
Switching MLLE3 with MLLE of PAB1 is an interesting approach. One might expect that Rrm4 can be recruited to endosomes again. However, Rrm4 would also interact with numerous other proteins containing PAM2 motifs like deadenylase Not4. Here it would compete with the MLLE of Pab1. Thus, it would be expected that Rrm4 is on the surface, but the protein will be mistargeted to other proteins causing pleiotropic alterations. It will be difficult to judge whether Rrm4 functionality is restored or whether other processes are disturbed. In essence, these are stimulating ideas, but we believe that these experiments are beyond the scope of the current study. In the future, we might address this point by using a heterologous peptide-binding pocket or tethering approach.

3. Likewise, what happens if Upa1 only has PAM2L2 instead of only PAM2L1 domains? Does that alter function - perhaps now one can observe a contribution of MLLE1? If it it's there it's likely to have function. Anything known about the post-translational modification of these MLLE or PAM domains? Does it change during unipolar vs. bipolar growth? Perhaps the different MLLE domains are regulated in such a fashion?
Again also very valid points. Upa1 with two PAM2L2 motifs might interact stronger. The problem is that one PAM2L motif is sufficient for interaction, and we do not see a strong phenotype.
Currently, we do not know if post-translational modifications regulate the MLLE domains. This could alter the binding affinity or specificity, and by expressing fungal proteins in *E. coli,* we might have missed this type of regulation. However, we addressed the function of MLLE1 and MLLE2 in *U. maydis* using a genetic approach. We deleted the corresponding domains and interfered with potential regulation by posttranslational modification. Thus, we cannot exclude post-translational modification, but it appears to be not essential for function. We will address the posttranslational regulation of Rrm4 in more detail in the future.

4. Can the authors show whether the binding of mRNA cargo (e.g. Cdc3 mRNA) to the RRM motifs of Rrm4 affects the interaction between any of the MLLE-PAM2L pairs, or vice versa (i.e. does the MLLE-PAM2L interaction affect mRNA binding)?
In previous studies, we have investigated a version of Rrm4 carrying a mutation in the first RRM motif of Rrm4. According to RNA live imaging, the respective strains exhibit a loss of function phenotype and mRNA transport is strongly affected. However, the endosomal association of Rrm4-mR1-Gfp is not affected, indicating no direct cross-talk between RNA-binding via RRM1 and endosomal attachment via MLLE3. Also, a version of Rrm4 carrying a deletion of all three RRM domains is still shuttling on endosomes. The two functions, i.e. RNA binding and endosomal binding, appears to be carried out by two independent platforms, i.e. three RRMs and three MLLEs, respectively. The overall structure of the protein also reflects this. The RRM domains are structurally clearly separated from the flexible MLLE domains.

5. Discussion line 311 It is written that the three MLLE domains "collaborate for optimal functionality..." Perhaps there's a misunderstanding here, but the authors show that MLLE3 domain alone is necessary & sufficient for function, so where is the collaboration? MLLE2 may have an accessory role according to the authors, but we do not know if it is in collaboration with MLLE3 or independent thereof. Since the KD of MLLE3 is not affected by the presence or absence of MLLE1,2 in vitro at least, it may be that they have independent, and not collaborative, roles.
Correct, we rephrased this more carefully. We omitted the collaboration aspect. It now reads, ”but a sophisticated binding platform consisting of three MLLE domains with MLLE2 and MLLE3 functioning in linking the key RNA transporter to endosomes.”

Reviewer #2 (Significance (Required)):
This paper concerns functional domains found in an endosome-localized RNA binding protein, U. maydis Rrm4, which is necessary for localized translation on endosomes and subsequent unipolar growth. Here the authors show using structural, biochemical, and genetic studies that instead of one or two MLLE protein-protein interacting domain in Rrm4 there are three, although one (MLLE3) is necessary and sufficient for full function. This work is for an audience interested in those studying RNA trafficking and its role in cell physiology, which is our expertise. The work is interesting, but it could be made more so especially if a connection was established between the RNA-binding function of the RRM domains and the MLLE-PAM2L interaction(s). At this point it is solid technical work and could be published after minor revisions.

****Referee Cross-commenting****
I concur with the comments of the other reviewers in that the work is solid and necessitates minor revisions in order to be published. Clearly, establishing a connection between the RNA-binding function and the MLLE-PAM interactions of Rrm4 would be an interesting and

worthy pursuit that might enhance the novelty of the work, but I agree that it could belong to future studies.

Reviewer #3 (Evidence, reproducibility and clarity (Required)):

Summary:
Long-distance subcellular transport of mRNAs is achieved through selective and dynamic interaction with the transport machinery. Using the highly polarized hyphae of Ustilago maydis, the authors previously showed i- that mRNAs can hitchhike on actively transported endosomes for proper distribution, and ii- that the connection between mRNAs and endosomes is mediated by the interaction between a C-terminal MademoiseLLE (MLE) domain of the RNA binding protein Rrm4 and the Upa1 adapter protein.
In this study, the authors aimed at more precisely characterizing the structural and molecular bases underlying the Rrm4-Upa1 interaction. Combining structural modeling and X-ray analyses, they discovered a non-canonical, and previously missed, MLE domain (MLE1) in Rrm4, and characterized the structure of the second MLE domains (MLE2) of Rrm4. Through binding assays, they showed that the three MLE domains exhibit different binding properties, and that MLE3 is the only domain capable of binding to the PAM2 domain of Upa1. Consistent with this finding, functional assays performed in U. maydis revealed that MLE3 is the main domain involved in interaction with endosomes and trafficking, MLE1 and 2 having either no or minor functions in this process.

The manuscript is very-well written, the data are of high quality and clearly presented. A wide range of complementary approaches has been used to molecularly and functionally characterize the different MLE domains of Rrm4. From an "RNA transport" perspective, this manuscript falls short of a main novel findings as the domains characterized in this study (MLE1 and 2) don't have a clear function in connecting mRNAs to the transport machinery. From an "MLE domain" perspective, this work however provides interesting information about non-canonical domains and structures, and about binding and function specificity.
As described below, my major concern relates to the role played by the ML2 domain of Rrm4, a role referred to as "accessory" by the authors.

Major comments:
The authors conclude from their results that ML2 has an accessory role in promoting association with endosomes.

1- This conclusion is made based on in vivo experiments showing that a form of Rrm4 lacking the M2 domain, in contrast to wild-type Rrm4, aberrantly attached to MTs in a context where the Rrm4-Upa1 interaction mediated by MLE3Rrm4 has been weakened (Upa1-pl2m). Although the results are convincing, their interpretation is less. The authors,

indeed, claim that the observed phenotype results from "the static accumulation of Rrm4" due to reduced interaction with endosomes. Why then don't they see a decrease in the motility/transport properties of Rrm4-M2Δ in this context then? Also, do the authors see a decrease in the co-localization of Rrm4-M2Δ with endosomes (which would be expected if the interaction is decreased)? Can the authors perform IP or co-sedimentation experiments to strengthen their hypothesis?

This is a fair criticism that was also raised by reviewer 1. In the improved version of the manuscript, we now include important control experiments demonstrating that (i) the aberrant localisation is microtubule-dependent (Fig. EV5F) (ii) the mutations do not cause differences in protein amounts of Rrm4 (Fig. EV5G) (iii) the key findings of the aberrant microtubule staining, which were scored manually in microscopic images were verified independently by two persons (Fig. EV5H) and (iv) most importantly, Rrm4 signal intensity is decreased in processive signals of our kymograph analysis (Fig. 5E). We firmly believe that this set of experiments strengthens our conclusion that MLLE2 plays an accessory role in the endosomal attachment (Fig. 6).

2- Whether MLE2Rrm4 mediates interaction with endosomes through association with Upa1 is unclear, as the binding assays performed in Figure 3 test for association of Rrm4 variants with single isolated domains of Upa1, not with the full-length protein. Assessing the binding of Rrm4-M2Δ variants with Upa1-PL2m would help interpreting the phenotypes described in Figure 5.

Unfortunately, it is difficult to express full-length Upa1 protein in *E. coli* due to the presence of extended unstructured regions. To overcome this limitation, we performed yeast two-hybrid experiments with full-length proteins of Rrm4 and Upa1. We were able to recapitulate qualitatively the results observed *in vitro* using the individual domains.

Notably, the Rrm4 version carrying a deletion in MLLE1 and MLLE2 interacted with Upa1 versions carrying mutations in PAM2L1 or PAM2L2 (Fig. EV3C), suggesting that both MLLE domains of Rrm4 are dispensable for interaction with Upa1. MLLE3 is sufficient to interact with a single PAM2L sequence of Upa1. This suggests the presence of additional interaction partners for MLLE1 and MLLE2 and is entirely consistent with our genetic and cell biological analysis described in Fig. 5.

Minor comments:

1- The authors have previously characterized the effect of a C-terminal deletion of Rrm4 on Rrm4 motility and binding to Upa1 (Becht et al., 2006; Pohlmann et al., 2015). How their previously-described construct compares to the Rrm4-M3Δ used in this study is unclear (is it the same?).

It is the identical mutation to allele *rrm4G*$^{P\Delta}$ from Becht et al. 2006. We indicate the information in the text "(Fig. 4B-C; mutation identical to allele *rrm4G*$^{P\Delta}$ in Becht *et al.*, 2006)."

2- page 6, line 141: refer to Fig. 1B rather than Fig. EV1A ?
We included the reference to Fig. 1B.

3- page 10, line 274: "Rrm4-Kat was found"
We corrected this.

4- page 11, line 286: "in strains expressing Upa1-PAM2L1", replace by "in strains expressing Upa1 with mutated PAM2L1"?
We corrected this.

5- The Figures and accompanying legends are overall very clear and detailed. In Figures EV4A and EV5D-E, it would however help if the authors would indicate on the Figure itself, left to each panel which markers/signals is being analyzed (e.g Rrm4-Kat (top) and Upa1-GFP (down) for Figure EV4).
We clarified this.

Reviewer #3 (Significance (Required)):
Active transport of mRNAs along microtubule tracks has been shown to play a key role in the spatio-temporal control of gene expression in various cell types and species. How specific mRNAs mechanistically connect to molecular motors for their transport to their subcellular destination has however for long remained largely unclear. Recent work, including work from the authors, has uncovered that RNAs can hitchhike on membranous organelles through adapter proteins linking mRNAs and RNA binding proteins with trafficking membrane-bound organelles.
This study aimed at investigating the structural and molecular bases underlying the interaction between RNA binding proteins and endosomes. While their identification and characterization of the MLE1 and MLE2 domains of Rrm4 did not provide significant new insight into the mechanisms involved in the endosome-mediated transport of mRNAs, it uncovered interesting new properties of MLE domains, including structural variations, selective binding and functional specificity. This work should thus be of interest for structural biologists and researchers interested in protein-protein interaction platforms.

****Referee Cross-commenting****
Our comments all converge to the idea that this study is solid as it is and requires only minor revision work to support the authors conclusions. Although characterizing further MLE/PAM2 binding specificity and MLE2 interactors would be of great interest and indeed provide a more complete understanding of interaction networks at play, I feel that this is beyond expected revision work.
