# Supplementary material for: A MademoiseLLE domain binding platform links the key RNA transporter to endosomes
Source: PLoS Genet. 2022 Jun 21;18(6):e1010269. doi: 10.1371/journal.pgen.1010269 (PMC9249222; doi:10.1371/journal.pgen.1010269)
Supplement: Attachment — Submitted filename: Response to Reviewers.pdf [file pgen.1010269.s022.pdf]

Dear Dr. Aaron P. Mitchell,

Thank you very much to accept our manuscript for publication in PLoS Genet. Please find enclosed our detailed description of the changes we made to the manuscript.

Reviewer's Responses to Questions

Comments to the Authors:
Please note here if the review is uploaded as an attachment.

Reviewer #1: The authors have satisfactorily addressed all my comments from the Review Commons evaluation process. I also believe they have done a good job of addressing the comments of the other reviewers. I now recommend publication provided the following very minor points are addressed:

1. On line 295 the authors conclude that the pattern in microtubule-related but only show the evidence (benomyl treatment) that this is the case later in this section. The authors should fix this, for example by first pointing out that the pattern was reminiscent of the microtubule association seen in the previous study and then confirming that the signal was derived from microtubules with benomyl.

We corrected this. The text now reads:

"When both PAM2L motifs were mutated, the Rrm4-Kat version is mislocalised and exhibited aberrant staining of filamentous structures in about 80% of hyphae (Fig 5C-D). This staining pattern was reminiscent of the microtubule association of Rrm4 previously observed during altered accumulation of static Rrm4-Kat in *upa1Δ* strains (S5C Fig; Jankowski *et al.*, 2019). Quantifying Rrm4-Kat signals exhibiting processive movement in kymographs revealed that strains exhibiting aberrant staining of filamentous structures resulted in reduced fluorescence (Fig. 5E) indicating fewer Rrm4-Kat versions on shuttling endosomes. As an important control, we treated the strains with the microtubule inhibitor benomyl, demonstrating that aberrant staining is microtubule-dependent (S5F Fig)."

2. Mislocalised is spelled incorrectly on line 294.
Corrected.

3. There are some inconsistencies with the tense that need fixing, e.g.:

Line 199. "However, when both PAM2L1,2 motifs were mutated, the interaction between the Upa1 and Rrm4 is lost….."

There are other examples of conflicting tenses that need resolving.

We correct this examples and others in the text. See the annotated version.

Reviewer #2: The manuscript by Devan et al. has already been reviewed by three individuals through the Review Commons process. All three reviewers considered that the work represents a significant advance, as it provides important new information

about MLLE and PAM interactions that is relevant to the broader field of endosome-mediated RNA trafficking. I concur with this opinion. The previous three reviewers agreed that experimental evidence for the role of MLLE2 in facilitating Rrm4 association with endoscopes needed to be strengthened. I consider that the new experimental data included in Figures 5 and EV5 satisfactorily address this concern. I also think that the various minor revisions requested by the previous reviews have been fully addressed.

Reviewer #3: Devan, Scott-Verdugo et al. performed a structure-function study characterizing the interaction between Rrm4 and Upa1, two proteins required for mRNA transport on early endosomes in Ustilago maydis. The authors use structural prediction, structural biology, and biochemistry to determine that Rrm4 contains three MLLE motifs, with MLLE3 being required for association with two PAM2-like motifs within Upa1. They then perform cell biology in Ustilago maydis and confirm that MLLE3 motif is required for Rrm4 association with motile early endosomes. Finally, they find that the MLLE2 motif of Rrm4 also plays a role in tethering Rrm4 to early endosomes. These findings demonstrate an adaptable binding platform between Rrm4 and Upa1, suggesting potential complex regulation of RNA transport on early endosomes.

This submission was received by PLOS Genetics as a Review Commons article and has already undergone revision following review from three reviewers (prior to this review). I read the submission and previous revisions. This is an interesting, well-written, thorough study. The previous reviews were adequately addressed and the article should be accepted as-is.

In addition, we corrected some misspelled words and we uploaded a new version of Figure EV2B, since we noticed an error in the graphical representation. The measured values in the submitted version were not indicated correctly, since the quantified data did not reach 100%.

We firmly believe that this revised version is now suitable for publication in PLoS Genet.

Thank you very much for your efforts in advance, sincerely yours, Michael Feldbrügge
